# Short or Long Antibiotic Regimens in Orthopedics

**DOI:** 10.1056/NEJMoa2506756

**Published:** 2026-09-16

**Authors:** Martin McNally, Maria Dudareva, Michelle Kumin, Werner Vach, Claire L. Scarborough, Matthew J. Cichero, Ricardo Sousa, Jamie Ferguson, David A. Stubbs, Bridget L. Atkins, Alex J. Ramsden, Bernadette C. Young, Andre D. Carvalho, Adrian R. Kendal, Abtin Alvand, Antony J.R. Palmer, Con L. Loizou, Ben Kendrick, Adrian H. Taylor, Amirul Islam, Michael J. Riste, Anand Pillai, Sunil K. Sharma, Jonathan Folb, Neal Jacobs, Simon C. Ellis, Aaron B.Y. Ng, Daniel Perez-Prieto, Irene K. Sigmund, Reinhard Windhager, Alexis D. Iliadis, Andrew J.F. Bing, Henry Wynn Jones, John Williams, Ben J. Ollivere, Volker Alt, Simon Warren, Neil E. Jenkins, Andreas Leithner, Harriet C. Hughes, Steve A. Jones, O. Martin Williams, Matthew Scarborough

**Affiliations:** 1 Bone Infection Unit, Nuffield Orthopaedic Centre, Oxford University Hospitals, United Kingdom; 2 Nuffield Department of Medicine, University of Oxford, United Kingdom; 3 Basel Academy for Quality and Research in Medicine, Switzerland; 4 Great Western Hospitals Foundation NHS Trust, United Kingdom; 5 Porto Bone and Joint Infection Group, ULS Santo António, Porto, Portugal; 6 Manchester University NHS Foundation Trust, United Kingdom; 7 Brighton and Sussex University Hospitals NHS Trust, United Kingdom; 8 Royal Liverpool and Broadgreen University Hospitals NHS Trust, United Kingdom; 9 Salisbury NHS Foundation Trust, United Kingdom; 10 Northumbria Healthcare NHS Foundation Trust, United Kingdom; 11 Mid-Yorkshire Hospitals NHS Trust, United Kingdom; 12 Institut Hospital del Mar d’Investigacions Mèdiques, Spain; 13 Medizinische Universität Wien, Austria; 14 Barts Health NHS Trust, United Kingdom; 15 the Robert Jones and Agnes Hunt Orthopaedic Hospital NHS Foundation Trust, United Kingdom; 16 Wrightington, Wigan and Leigh NHS Foundation Trust, United Kingdom; 17 South Tees Hospitals NHS Foundation Trust, United Kingdom; 18 Nottingham NIHR Biomedical Research Centre, School of Medicine, University of Nottingham, United Kingdom; 19 University Hospital Regensburg, Germany; 20 Royal National Orthopaedic Hospital NHS Trust, United Kingdom; 21 University Hospitals Birmingham NHS Trust, United Kingdom; 22 Medizinische Universität Graz, Austria; 23 University Hospital of Wales, United Kingdom; 24 Cardiff & Vale University Health Board, United Kingdom; 25 University Hospitals Bristol NHS Foundation Trust

## Abstract

**Background:**

Orthopedic infection is usually managed with surgery and prolonged systemic antibiotic therapy. Whether local antibiotics delivered by a product implanted at the infection site during surgery can reduce the duration of systemic antibiotic therapy is unclear.

**Methods:**

In a multicenter, open-label, noninferiority trial, we randomly assigned, in a 1:1 ratio, adults who had undergone surgery for orthopedic infection and implantation of a local-antibiotic carrier to receive postoperative systemic antibiotic therapy for a long (≥4 weeks) or short (≤7 days) duration. The primary outcome was definite treatment failure by 12 months, assessed according to protocol-defined criteria by a clinical end-point committee whose members were unaware of the trial-group assignments. The noninferiority margin was 10 percentage points.

**Results:**

A total of 500 patients underwent randomization, of whom 475 were assessed in the primary analysis. Definite treatment failure occurred in 34 of 241 patients (14.1%) in the long-duration group and in 26 of 234 patients (11.1%) in the short-duration group (risk difference [negative values favor the short duration], −3.0 percentage points; 95% confidence interval [CI], −9.0 to 3.0), which met the prespecified noninferiority margin. In sensitivity analyses of the randomized population (497 patients) and the per-protocol population (457 patients), results were consistent with those of the primary analysis (risk difference, −2.4 percentage points [95% CI, −8.2 to 3.5] and −2.7 percentage points [95% CI, −8.8 to 3.4], respectively). By week 6 after surgery, symptoms potentially related to treatment had occurred in 45.2% of the patients in the long-duration group and in 17.2% of those in the short-duration group (risk difference, −28.0 percentage points; 95% CI, −36.4 to −19.6).

**Conclusions:**

Among patients who had undergone orthopedic surgery and received local antibiotics, a short course of systemic antibiotics was noninferior to a long course with respect to definite treatment failure by 12 months. (Funded by the European Bone and Joint Infection Society and others; SOLARIO ClinicalTrials. gov number, NCT03806166.)

The management of bone and joint infections, such as osteomyelitis, fracture-related infection, prosthetic joint infection, and diabetic foot infection, ordinarily includes a combination of surgical débridement and prolonged administration of systemic antibiotic therapy.^1–4^ The standard of care for the duration of systemic antibiotic therapy is generally at least 4 weeks.

Local antibiotics delivered by a licensed carrier that has been implanted during surgery are increasingly being used alongside systemic antibiotics in the treatment of orthopedic infection.^5–11^ Theoretical advantages of local antibiotics include delivery of very high antibiotic concentrations locally, limited systemic absorption of antibiotics and potentially fewer side effects, and improved antimicrobial stewardship.^10–14^ Local antibiotics may be particularly valuable in patients with impaired peripheral vasculature, poor adherence, or reduced gut absorption. However, local therapy may be associated with an increased risk of hypercalcemia, renal impairment, or local problems with wound leakage or fracture of an antibiotic-loaded spacer.^6,7,10,13^ We conducted the SOLARIO (Short or Long Antibiotic Regimes in Orthopaedics) trial to investigate whether the use of local antibiotic therapy could safely facilitate a large reduction in the duration of systemic antibiotic treatment in patients with bone and joint infection.

## Methods

### Trial Design and Oversight

The current trial was an open-label, randomized, noninferiority trial conducted at 25 sites in the United Kingdom,{q3} Portugal, Spain, Austria, and Germany. All participants provided written informed consent. The primary end point was adjudicated by members of an independent clinical end-point committee who were unaware of trial-group assignments. The trial was approved by the South-Central Research Ethics Committee on behalf of the U.K. Health Research Authority. The trial protocol was published previously^15^ and is available with the full text of this article at NEJM.org.

### Patients

The trial recruited adults (≥18 years old) who were to undergo definitive (curative) surgery for a confirmed orthopedic infection and would ordinarily have received systemic antibiotic therapy for at least 4 weeks after surgery. During surgery, all patients underwent deep-tissue sampling, excision of dead bone, removal of infected metalware and prostheses, implantation of licensed local-antibiotic carrier, and closure of the surgical wound. The surgeon could choose internal or external fixation of the fracture in patients with fracture-related infection or osteomyelitis with an indication for stabilization after débridement. Prosthetic-joint{q4} infection was managed with single-stage revision involving an antibiotic-loaded cement product or with removal of the prosthesis and implantation of an antibiotic-loaded spacer, with or without antibiotic-eluting pellets.

Patients who had undergone noncurative surgery (i.e., procedures to reduce the bioburden and administer suppressive antibiotic therapy), repeated débridement, or a débridement, antibiotics, and implant retention (also known as DAIR) procedure were excluded (additional details are provided in the Supplementary Appendix, available at NEJM.org).^15^ Patients with negative cultures were eligible for inclusion in the trial, but those with bacteria that were fully resistant to the local antimicrobial therapy were excluded.

### Randomization and Treatment

Within 7 days after surgery, patients were assigned, in a 1:1 ratio, to receive postoperative systemic antibiotic therapy for a short duration (≤7 days) or a long duration (≥4 weeks). The duration of systemic therapy was quantified according to an international{q5} multidisciplinary consensus procedure.^16^ Randomization was conducted by means of a customized clinical database management system^17^ with the use of permuted blocks of 2 to 12 and stratification according to trial site.

During surgery, all the patients underwent implantation of a licensed local-antibiotic carrier that eluted high-concentration antimicrobials at the infection site for at least 4 weeks. The local and systemic antimicrobial regimens were not dictated by the trial protocol but were typically chosen by an infectious-disease specialist at the trial site on the basis of clinical and microbiologic data and local epidemiologic data.

### Outcomes

The primary outcome was definite treatment failure within 12 months after surgery. Definite treatment failure was defined by the presence of at least one of the following features or events as assessed by the clinical end-point committee: a sinus tract or purulence at the surgical site, infection as detected by microbiologic or histologic analysis (or both) at reoperation, newly elevated levels of biomarkers in synovial fluid at the time of aspiration or reoperation, or death from infection at the index surgical site within 12 months after surgery. The primary objective was to assess the noninferiority of a short course of postoperative systemic antibiotic therapy to a long course with respect to the primary outcome.

Secondary outcomes included potential side effects of antibiotic therapy, duration of hospital stay, serious adverse events, overall survival, and quality of life. Additional details about the primary and secondary outcomes are provided in the Supplementary Appendix.

### Data Collection and Assessments

Baseline data were recorded at enrollment. Participants were followed up during scheduled visits, which were to occur at 6 weeks, anytime between 3 and 6 months, and at 12 months after surgery. When patients were unable to attend appointments, telephone review, contact with the patient’s primary care team, and health records were used to identify potential treatment failures, side effects of antibiotic therapy, and serious adverse events.

The clinical end-point committee was sent a clinical summary, redacted for information that might reveal the trial-group assignment, when any of the following features of potential treatment failure were present: any hospital readmission, unplanned operation, wound dehiscence or new sinus formation, prescription of new antibiotics, death, or any clinical concern from the patient’s treatment team. The clinical end-point committee distinguished between definite, probable, possible, and no treatment failure according to prespecified criteria (see the Supplementary Appendix for details).

At each follow-up visit, patients were asked to report whether nausea, vomiting, diarrhea, lethargy, or rash related to antibiotic use had occurred since the previous visit. All five symptoms had been chosen for assessment by patient representatives during a focus group that had been convened at the time of trial design. The{q6} treating clinicians actively monitored for surgical wound leakage and *Clostridioides difficile* infection at all visits, including those scheduled at 6 weeks, 3 to 6 months, and 12 months. Patients were also encouraged to report any other symptoms related to treatment{q7}. The severity of the event was assessed{q8} according to prespecified categories as mild to moderate (with no indication for treatment) or severe, frequent, or having an indication for treatment.

Serious adverse events, as defined according to criteria available at ClinicalTrials.gov,^18^ were recorded during all{q9} visits at each site for up to 1 year after the index surgery. The duration of hospitalization and overall survival were determined on the basis of data from electronic records. Quality of life as reported by the patient was measured at enrollment and 12 months after surgery with the EuroQol Group 5-Dimension 5-Level (EQ-5D-5L) questionnaire.^19^ Scores on the utility index of the EQ-5D-5L range from less than 0 to 1, with 0 indicating death, 1 full health, and negative values health states considered to be worse than death. Scores on the visual analogue scale (VAS) of the EQ-5D-5L range from 0 to 100, with 0 indicating worst imaginable health and 100 best imaginable health.

## Statistical Analysis

The noninferiority margin for the primary outcome was set at 10 percentage points. This margin has been used in previous studies of bone infection^20–22^ and was supported by the patient focus group.^15^ We estimated that a sample size of 492 patients would provide the trial with a power of 90% to show that a short duration of systemic antibiotic therapy was noninferior to a long duration, using a one-sided test at the 2.5% significance level. This calculation assumed that definite treatment failure would occur by 12 months in 13% of the trial population.^4,9,15^ The sample size of 492 was rounded up to 500 before enrollment.

Patients who were lost to follow-up (those who could not be reached by the trial investigators and had no contact information for their general practitioner) were assumed not to have had a primary outcome event (see the Supplementary Appendix). The treatment effect is reported as a risk difference with a 95% confidence interval, with a negative risk difference favoring the short duration of antibiotics. A one-sided test at the 2.5% significance level was conducted to assess the null hypothesis that the risk difference would exceed the noninferiority margin.

Three populations were considered in the analyses. The randomized{q10} population included all the patients who had undergone randomization. The eligible population included all the patients who had undergone randomization and met the postrandomization inclusion criteria{q11}. The per-protocol population included all the patients in the eligible population except those in the long-duration group who had received systemic antibiotics for less than 28 days and those in the short-duration group who had received systemic antibiotics for more than 7 days. The primary analysis was conducted in the eligible population. The randomized and per-protocol populations were assessed in sensitivity analyses only. Sensitivity analyses were also performed with the alternative definitions of treatment failure (definite treatment failure, definite or probable treatment failure, and definite, probable, or possible treatment failure; see the Supplementary Appendix).

Symptoms related to antibiotic treatment other than the five prespecified symptoms were summarized into one additional variable that reflected the worst severity level reported by the patient. The number of episodes of each symptom are reported for each follow-up time point.

Serious adverse events, excluding treatment failure, are reported as the number and percentage of patients with the event and as the number of events per patient. Overall survival is reported as the percentage of patients who were alive at month 12. The hazard ratio for the between-group difference in overall survival was calculated with a Cox proportional-hazards model. The length of the hospital stay is summarized with means and 10th and 90th percentiles. Quality of life as reported by the patient was assessed as the mean change in the utility index score from baseline to month 12 and as the mean VAS score at 12 months.

Unless otherwise specified, treatment effects were assessed as the risk difference or difference in means with their 95% confidence interval. The widths of the confidence intervals have not been adjusted for multiplicity and should not be used in place of hypothesis testing.

Prespecified subgroup analyses were performed to assess for heterogeneity in the treatment effect according to baseline demographic and clinical characteristics and indications for surgery. In post hoc analyses, the primary outcome was assessed with regard to osteomyelitis and fracture-related infection combined, prosthetic joint infection, and *Staphylococcus aureus* infection. Deviations from the trial protocol are described in the Supplementary Appendix.

## Results

### Patients

From February 2019 through August 2023, we assessed 1211 patients for eligibility, of whom 500 were recruited into the trial and underwent randomization. Of the 711 patients who were excluded, 21 had an indication for systemic antibiotics for other reasons (e.g., sepsis or endocarditis). After 100 patients had{12">q12} completed the 12-month follow-up period, the data and safety and monitoring board undertook an unblinded analysis of the safety of the trial. No unexpected safety events were identified.

**Figure 1:**
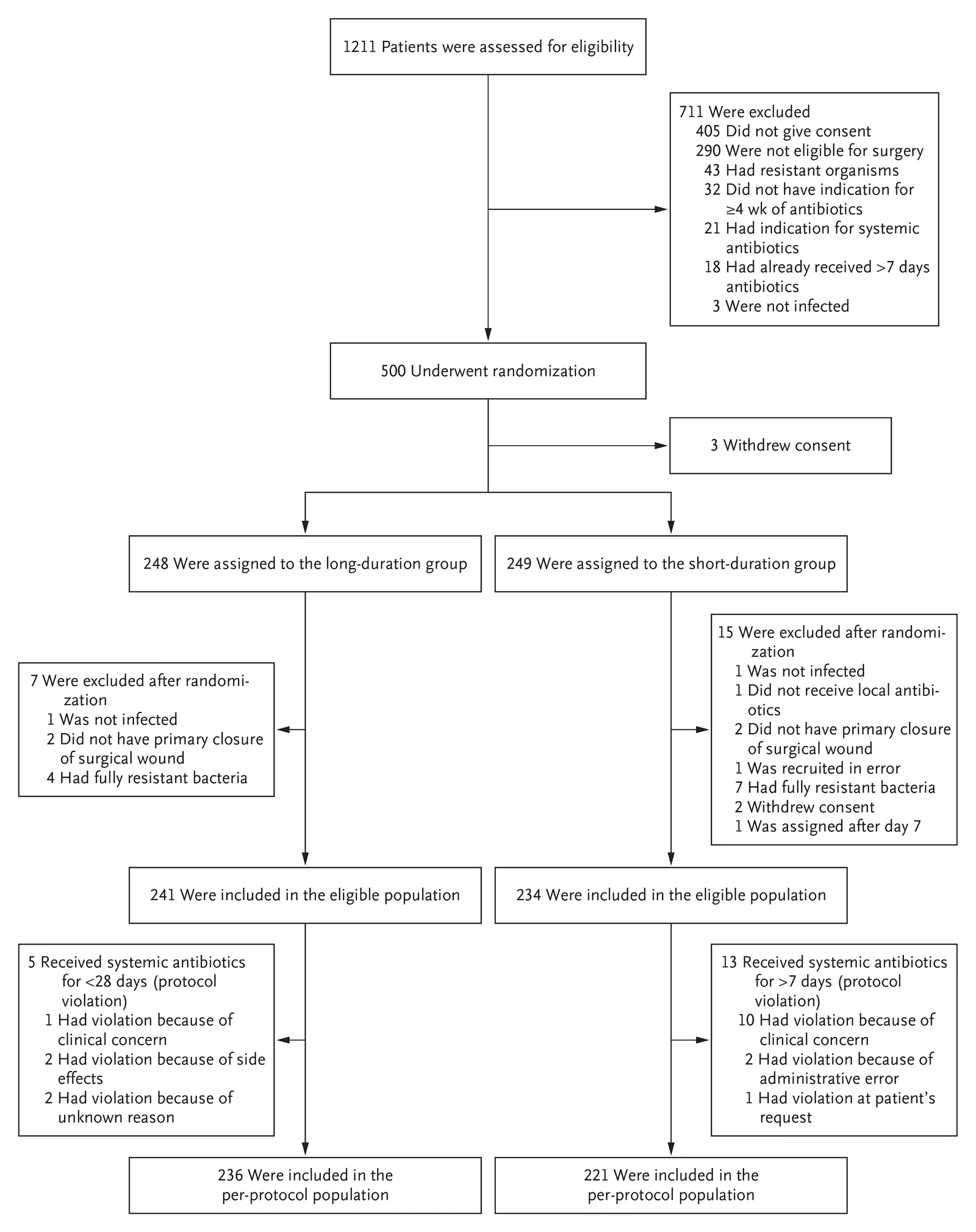
Trial Populations. Adults who had undergone surgery for orthopedic infection and implantation of a licensed antibiotic-carrier product at the infection site were assigned to receive a long duration (≥4 weeks) or short duration (≤7 days) of systemic antibiotic therapy after surgery. The randomized population included all the patients who were eligible for randomization except for three patients who withdrew consent for ongoing use of their data. The eligible population included all the patients who had undergone randomization and met the postrandomization inclusion criteria. The per-protocol population included all the patients in the eligible population except those in the long-duration group who had received postoperative systemic antibiotics for less than 28 days and those in the short-duration group who had received postoperative systemic antibiotics for more than 7 days. DAIR denotes débridement, antibiotics, and implant retention.

The randomized population included 497 patients after the exclusion of 3 patients who withdrew consent for ongoing use of their data (Fig. 1). An additional 22 patients were excluded after randomization because they did not meet eligibility criteria, leaving 475 patients in the eligible population. There were 18 protocol violations regarding the duration of postoperative antibiotic therapy, leaving 457 participants in the per-protocol population. Three patients in the long-duration group and 8 patients in the short-duration group (2.3% of the eligible population) were lost to follow-up for the primary outcome.

In the randomized population, baseline characteristics of the patients and indications for surgery were well matched between the trial groups (Table 1 and Table S5). Overall, 85.3% of the patients had undergone multiple previous operations at the same anatomical site and 80.3%{q13} had a complex coexisting condition (American Society of Anesthesiologists physical status classification of grade 2 or higher){q14}. A total of 175 patients (35.3%){q15} underwent débridement for osteomyelitis or fracture-related infection, 159 (32.1%) underwent débridement for osteomyelitis or fracture-related infection with removal or exchange of an implant, and 96 (19.4%) underwent single-stage or two-stage treatment of prosthetic joint infection. All the patients underwent closure of the surgical wound, and 90 patients (18.3%){q16} underwent complex soft-tissue reconstruction (wound closure with a local or free flap). The representativeness of the trial population is shown in Table S1.

### Systemic and Local Antimicrobial Therapy

The median duration of postoperative systemic antibiotic therapy was 6 days (interquartile range, 5 to 7) in the short-duration group and 42 days (interquartile range, 41 to 48) in the long-duration group. Local antibiotic therapy was delivered by an implanted absorbable carrier in 389 patients, by an implanted polymethylmethacrylate (PMMA)–based carrier in 66 patients, and by an implanted absorbable product and an implanted PMMA-based product in 38 patients. Details about the local antibiotics and carriers are provided in Tables S2 and S3.

Eleven patients (2.2%) were excluded from the trial because culture of tissue obtained from the surgical site grew bacteria that were fully resistant to local antibiotics. Pathogen-specific systemic therapy was selected by an infectious-disease specialist on the basis of culture results or local policy when cultures were negative.

### Primary Outcome

Among the patients in the eligible population, definite treatment failure occurred in 26 of 234 (11.1%) in the short-duration group and in 34 of 241 (14.1%) in the long-duration group (risk difference, −3.0 percentage points; 95% confidence interval [CI], −9.0 to 3.0), which showed the noninferiority of the short duration. Overall, 41 patients (68.3%) with definite treatment failure presented with a new draining sinus (Table S4); failure was usually confirmed by means of microbiologic and histologic analysis after repeat surgery. One{q17} patient (1.7%) had definite treatment failure diagnosed on the basis of increased levels of synovial inflammatory markers alone.

**Figure 2:**
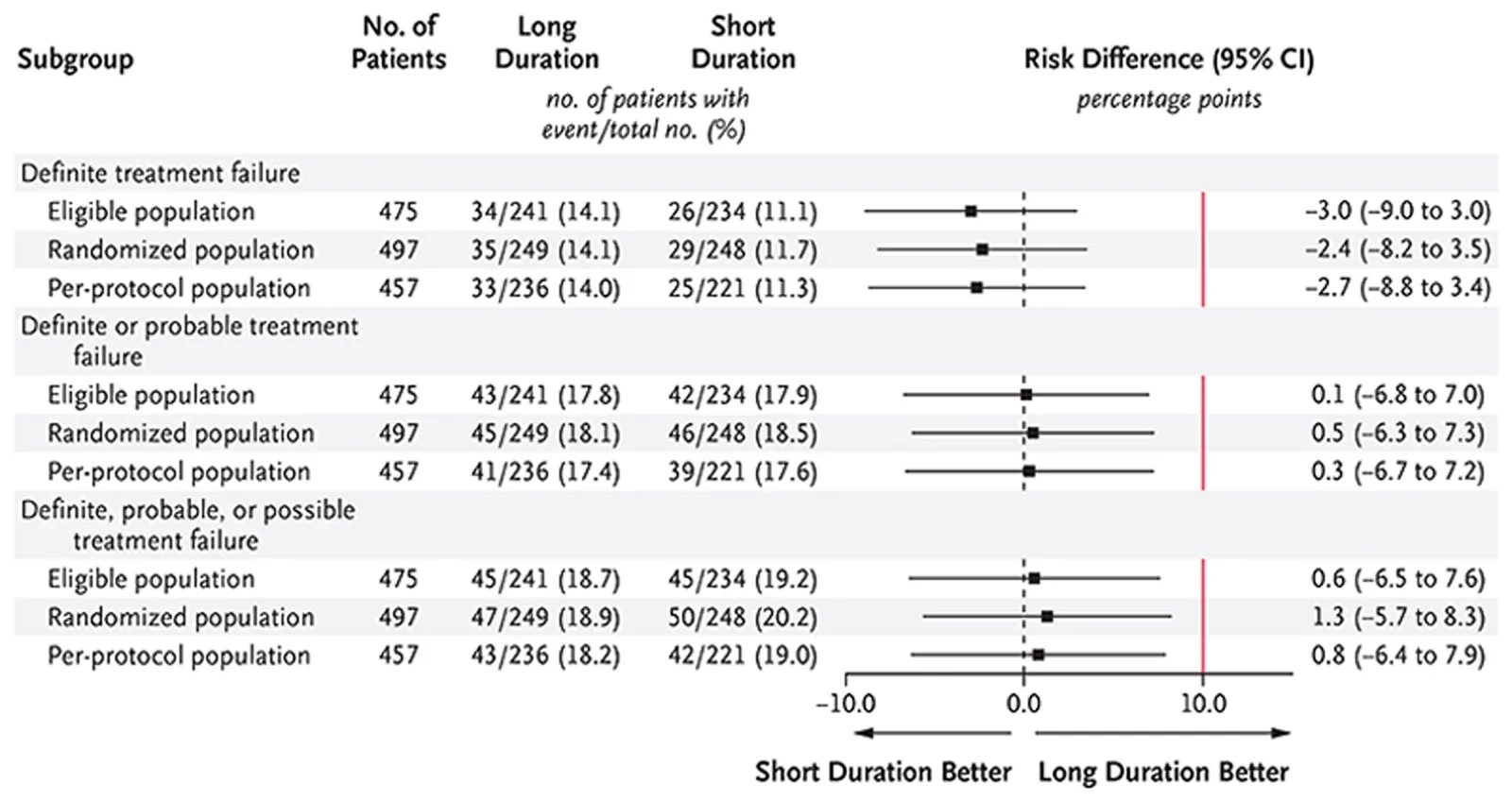
Robustness of the Primary Analysis Results. Shown is the robustness of results of the primary analysis to alternative definitions of treatment failure and the three analysis populations. The primary outcome was definite treatment failure within 12 months after surgery. The primary analysis population was the eligible population. Definite treatment failure was defined by the presence of at least one of the following features or events: a sinus tract or purulence at the surgical site, detection of infection by microbiologic or histologic analysis (or both) at reoperation, newly elevated levels of biomarkers in synovial fluid at the time of aspiration or reoperation, or death from infection at the index surgical site within 12 months after surgery, as confirmed by the independent clinical end-point committee. Definitions of probable and possible treatment failure are provided in the Supplementary Appendix. The red line indicates the noninferiority margin. The widths of the confidence intervals have not been adjusted for multiplicity and should not be used in place of hypothesis testing.

**Figure 3:**
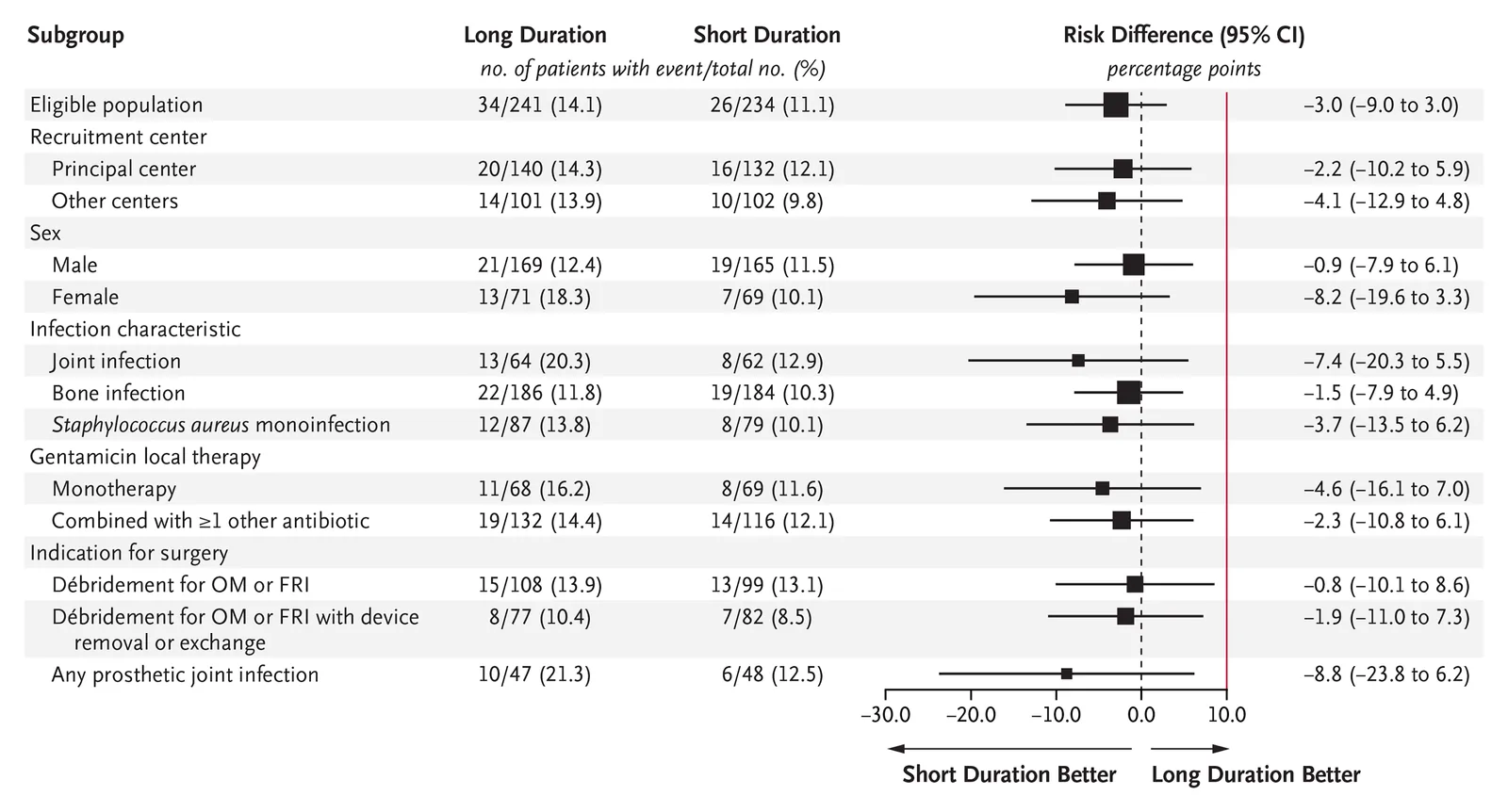
Subgroup Analyses. Shown{q22} are prespecified subgroup analyses to explore the heterogeneity in treatment effect according to baseline demographic and clinical characteristics and indications for surgery. The Oxford Bone Infection Unit was the principal recruitment center. The size of the squares is proportional to the number of patients in each subgroup. The red line indicates the noninferiority margin. The widths of the confidence intervals have not been adjusted for multiplicity and should not be used in place of hypothesis testing. FRI denotes fracture-related infection, and OM osteomyelitis.

In sensitivity analyses, results of the primary analysis were robust to the alternative definitions of treatment failure and the three patient populations (Fig. 2 and Figs. S1 and S4 through S6). The risk difference was close to zero in all the analyses and therefore within the noninferiority margin. Prespecified subgroup analyses showed no substantial heterogeneity in treatment effect, and the results were consistent with those of the primary analysis (Fig. 3).

### Secondary Outcomes

Symptoms potentially related to antibiotic treatment were reported at the 6-week follow-up visit by 45.2% of the patients in the long-duration group and 17.2% of those in the short-duration group (risk difference, −28.0 percentage points; 95% confidence interval, −36.4 to −19.6). Such symptoms were reported at the visit between 3 and 6 months by 20.4% and 8.5%, respectively (risk difference, −12.0 percentage points; 95% CI, −19.2 to −4.7) (Table 2 and Fig. S3).

Mild-to-moderate symptoms and severe symptoms generally occurred in fewer patients in the short-duration group than in the long-duration group (Table S8). *C. difficile* infection was diagnosed in two patients in the long-duration group; both infections were severe and occurred within 6 weeks after surgery.

The mean duration of the hospital stay was 14.8 days in the long-duration group and 11.9 days in the short-duration group (mean difference, −2.9; 95% CI, −7.0 to 1.2) (Table 2). Quality of life as assessed with the EQ-5D-5L questionnaire was similar in the trial groups. The mean (±SD) change in the utility index score from baseline to month 12 was 0.13±0.28 in the long-duration group and 0.12±0.27 in the short-duration group, and the mean VAS score at month 12 was 70.93±20.83 and 71.65±21.82, respectively.

Serious adverse events occurred in 48 patients in the long-duration group and 40 patients in the short-duration group; death occurred in 9 patients and 5 patients, respectively (Table 2 and Tables S6 and S7). Overall survival at 12 months was 96.3% in the long-duration group and 97.9% in the short-duration group (hazard ratio for death, 1.76; 95% CI, 1.21 to 5.42) (Table 2 and Fig. S2).

## Discussion

This trial challenges the standard of care for orthopedic infection, which usually includes at least 4 weeks of postoperative systemic antibiotic treatment. Treatment with local antibiotics delivered by a carrier implanted during surgery allows patients and clinicians to consider a shorter duration of systemic antibiotic regimen after surgery, but the shorter regimen must not be associated with an increased risk of failure.

Participation in the current trial was limited to patients who had undergone implantation of a licensed antibiotic carrier. These carriers included PMMA cement, calcium sulfate, and hydroxyapatite ceramic products that can deliver antibiotics for at least 4 weeks with well-defined elution profiles.^11,14,23,24^ The carriers were composed of absorbable or nonabsorbable materials, which have different elution characteristics. In general, the use of carriers with a definite release time is preferred to avoid prolonged low-level elution, which may exert a selection pressure on residual organisms. Patients who received raw antibiotic powder sprinkled into the operative site, irrigation devices, or intraosseus infusions were not included in the trial. The results of the current trial should not be used to support the use of these techniques for delivery of local antibiotics.

The addition of local antibiotic therapy to systemic antibiotic therapy has been associated with a lower risk of treatment failure in cohort and registry studies of bone and joint infection.^5–9,25–27^ There is precedence in observational studies for relying primarily on local antibiotic therapy in patients with orthopedic infection,^28,29^ However,{q18}.

Side effects of systemic antibiotic therapy are a major healthcare burden.^4,30–33^ The anticipated advantages of short-duration therapy, which include fewer symptoms associated with treatment and shorter durations of hospital stay without an increased risk of death or serious adverse events, were seen in the current trial. Local antimicrobial therapy also removes concerns about adherence. The use of shorter-duration systemic antibiotic therapy and the limited systemic absorption of antibiotics that are delivered at the surgical site^10,14,34^ have favorable implications for antimicrobial stewardship owing to reduced selection pressure on the microbiome. Nonetheless, the use of broad-spectrum systemic therapy during the immediate perioperative period remains necessary for prophylaxis against surgical-site contamination or transient bacteremia during surgery and to allow time to test for antibiotic resistance.

Subgroup analyses did not show clinically relevant heterogeneity. There was no subgroup for which the estimate of definite treatment failure at 12 months varied greatly from the estimate for the entire trial population. This is important because the trial had pragmatic inclusion criteria, which resulted in the inclusion of patients with a representative range of bone and joint infections, including complex and multisite infections. Among the 95 patients who had received a new metal implant during the surgical procedure for orthopedic infection, there was no increased failure rate in the short-duration group, which suggests that the local antibiotics had protected the new implant from colonization.

Formal health economics analyses of the trial data are being conducted. Direct costs will likely be lower with the short duration than with the long duration given that the short-duration group used fewer systemic antibiotics and had a shorter length of hospital stay and lower number of events with an indication for treatment.

Our trial has potential limitations. The trial had an open-label design because it was not feasible to produce placebos to match the broad range of oral and intravenous antibiotics that were prescribed. We limited the risk of consequent bias by using prespecified outcome definitions and an independent clinical end-point committee that was unaware of trial-group assignment and antibiotic use. Patients who had undergone a débridement, antibiotics, and implant retention procedure were excluded because such surgery is often not planned as a curative procedure, it is difficult to ensure the quality of the débridement, and penetration of local antibiotics into the infected area in this context is unknown. Thus, the findings should not be extrapolated to patients who are undergoing this procedure. The{q19} potential for selection bias exists given that clinicians with a more conservative approach to surgery for bone and joint infection may not have recruited patients with a high risk of treatment failure. However, the percentage of patients with definite treatment failure in the current trial is similar to that in other cohort studies. In addition, 85.3% of the patients in our trial had undergone multiple previous operations at the same anatomical site, 80.3% had a complex coexisting condition, and 18.4% had an indication for complex soft-tissue reconstruction. Only 21 of the 1211 patients (1.7%) assessed for trial eligibility were considered to be ineligible because they had an indication for a longer duration of systemic antibiotics (e.g., sepsis or endocarditis).

This trial was not designed to evaluate any particular combinations of local antibiotics and carriers. We investigated the strategy of relying primarily on local antibiotic therapy and secondarily{q20} on long-duration systemic therapy. The current trial was not recognized in the context of U.K. and European Union regulatory frameworks as a clinical trial of an investigational medicinal product. Therefore, the choice of local antibiotics was restricted to those included in antibiotic–carrier combinations that had been licensed for use during surgery at the time of ethics approval. Seventeen different licensed antibiotic–carrier combinations were used during the trial, which reflects the breadth of current clinical practice in Europe.

In the current trial, a short course (≤7 days) of systemic antibiotics was noninferior to a long course (≥4 weeks) with regard to definite treatment failure at 12 months in patients who had undergone surgical management of bone and joint infection that included local antibiotics.{q21}

## Supplementary Material

supplement

## Figures and Tables

**Table 1:** Baseline Characteristics of the Patients (Randomized Population).*

Characteristic	Long Duration	Short Duration
**Demographic characteristic**		
Female sex — no./total no. (%)	73/248 (29.4)	71/247 (28.7)
Median age at surgery (IQR) — yr†	59 (49–69)	57 (44–68)
**Clinical characteristic**		
Median Charlson Comorbidity Index score (IQR) §	2 (1–4)	2 (0–4)
Median body-mass index (IQR)§	28.7 (25.0–33.3)	27.8 (23.8–32.1)
Diabetes mellitus — no./total no. (%)	60/249 (24.1)	54/248 (21.8)
Presence of complications — no./total no. (%)¶	26/244 (10.7)	16/240 (6.7)
Current smoker — no./total no. (%)	44/244 (18.0)	43/245 (17.6)
Bone infection — no./total no. (%)	193/249 (77.5)	196/248 (79.0)
Joint infection — no./total no. (%)	66/249 (26.5)	63/248 (25.4)
>1 Previous operation at same anatomical site — no./total no. (%)	212/247 (85.8)	206/243 (84.8)
**Indication for surgery — no./total no. (%)**		
Debridement for osteomyelitis or fracture-related infection	87/249 (34.9)	88/247 (35.6)
Debridement for osteomyelitis or fracture-related infection with device removal or exchange{q23}	75/249 (30.1)	84/247 (34.0)
Debridement for diabetic foot osteomyelitis	31/249 (12.4)	20/247 (8.1)
Complex septic arthritis	9/249 (3.6)	5/247 (2.0)
Removal or first-stage revision of prosthetic joint	24/249 (9.6)	27/247 (10.9)
Single-stage revision of prosthetic joint	23/249 (9.2)	22/247 (8.9)
Complex soft-tissue reconstruction{q24}	45/246 (18.3)	45/242 (18.6)
Placement of new metal implant	47/249 (18.9)	51/247 (20.6)
Single-stage revision for prosthetic joint infection{q25}	24/249 (9.6)	22/247 (8.9)
Fracture-related infection with new internal fixation	23/249 (9.2)	29/247 (11.7)
**Infecting organism — no./total no. (%)**		
*Staphylococcus aureus*	88/249 (35.3)	84/248 (33.9)
*Staphylococcus epidermidis*	26/249 (10.4)	27/248 (10.9)
Pseudomonas species	12/249 (4.8)	10/248 (4.0)
>1 Organism	65/249 (26.1)	52/248 (21.0)

* Adults who had undergone surgery for orthopedic infection and implantation of a licensed antibiotic carrier at the infection site were assigned to receive a long duration (≥4 weeks) or short duration (≤7 days) of systemic antibiotic therapy after surgery. The randomized population included all the patients who had undergone randomization except for three patients who withdrew consent for ongoing use of their data. Complete baseline characteristics are provided in Table S5. IQR denotes interquartile range.

† Data are for 249 patients in the long-duration group and 248 patients in the short-duration group.

‡ Scores{q26} on the Charlson Comorbidity Index range from XXX to YYY, with higher scores indicating a greater burden of coexisting conditions. Data are for 249 patients in the long-duration group and 246 patients in the short-duration group.

§ Body–mass index is calculated as the weight in kilograms divided by the square of the height in meters. Data are for 245 patients in the long-duration group and 246 patients in the short-duration group.

¶ Data are for vascular, neurologic, and renal complications.

**Table 2:** Secondary Outcomes.*

Outcome	Long Duration	Short Duration	Treatment Effect (95% CI) †
Potential side effects of antibiotic therapy — no./total no. (%)‡			
Any symptom reported at 6 wk	98/217 (45.2)	35/204 (17.2)	–28.0 (–36.4 to –19.6)
Any symptom reported at 3–6 mo	37/181 (20.4)	14/165 (8.5)	–12.0 (–19.2 to –4.7)
Surgical wound leakage 1–6 wk after surgery	13/249 (5.2)	18/248 (7.3)	1.42 (0.68 to 2.97)
Mean length of hospital stay (10th to 90th percen-tile) — days§	14.8 (5.0 to 28.0)	11.9 (4.0 to 20.0)	–2.9 (–7.0 to 1.2) ¶
Serious adverse events‖			
Any event — no. (%)	48 (19.9)	40 (17.1)	–2.8 (–9.8 to 4.2)
Mean no. of events per patient	0.29	0.21	–0.07 (–0.18 to 0.04) ¶
Overall survival at 12 mo — %**	96.3	97.9	1.76 (1.21 to 5.42) ††
Quality of life‡‡			
Change from baseline to 12 mo in utility index score	0.13+0.28	0.12+0.27	–0.01 (–0.06 to 0.05) ¶
Visual analogue scale score at 12 mo	70.93+20.83	71.65+21.82	0.7 (–3.5 to 5.0) ¶

* Plus–minus values are means ±SD.

† Unless otherwise indicated, the treatment effect is reported as a risk difference, presented as percentage points, with a negative risk difference favoring the short duration of antibiotic therapy. The widths of the confidence intervals have not been adjusted for multiplicity and should not be used in place of hypothesis testing.

‡ Patients were asked to report whether any of the following five symptoms related to antibiotic use had occurred: nau-sea, vomiting, diarrhea, lethargy, or rash (see Table S8 for additional details). The treating clinicians actively looked for surgical wound leakage at all visits, including those scheduled at 6 weeks, 3 to 6 months, and 12 months. Leakage of the surgical wound during the first 6 weeks after surgery may be related to dissolution of an absorbable antibiotic carrier.

§ Data were available for 239 patients in the long-duration group and 231 patients in the short-duration group.

¶ Shown is the difference in means (short duration vs. long duration).

‖ Data exclude treatment failure and were available for 241 patients in the long-duration group and 234 patients in the short-duration group. Additional details about serious adverse events are provided in Table S6.

** Data were available for 241 patients in the long-duration group and 234 patients in the short-duration group. Details about the cause and time of death are provided in Table S7.

†† Shown is the hazard ratio for death, which favored the short duration of antibiotic therapy.

‡‡ Quality of life as reported by the patient was assessed according to the utility index score and the visual analogue scale score on the EuroQol Group 5-Dimension 5-Level questionnaire. Scores on the utility index range from less than 0 to 1, with 0 indicating death, 1 full health, and negative values health states considered to be worse than death; data were available for 208 patients in the long-duration group and 193 patients in the short-duration group. Scores on the visual analogue scale range from 0 to 100, with 0 indicating worst imaginable health and 100 best imaginable health; data were available for 201 and 188, respectively.

## Data Availability

A data sharing statement provided by the authors is available with the full text of this article at NEJM.org.

## Queries

q1 AU: (A) We have added initials as requested. Please note that we have not modified surnames, apart from insertion of the first initial for Dr. Williams. (B) We have added additional credentials as requested. Please note that we do not indicate lower degrees (e.g., M.Sc. and M.A.) for authors with doctoral (and equivalent) degrees. Also, some credentials may be omitted, pending interval review of proof 2, because they do not accord with our style. (C) Please note that affiliations and references will be revised per our style by our proofreaders during internal review of proof 2.

q3 AU: Is the order of countries relevant? If not, we will list in alphabetical order per our style.

q4 AU: Sentence OK?

q5 AU: Correct as edited? This does not appear to be an official name.

q6 AU: Sentence OK?

q7 AU: Would it be appropriate to remove “related to treatment” per your response to Q21 in proof 1?

q8 AU: OK as edited?

q9 AU: “all visits” OK?

q10 AU: Our style does not permit “randomized population” or “randomized patients.” Is there another term, such as “modified intention-to-treat population” that can be used instead of “randomized population”?

q11 AU: OK to stet, given the sentence beginning “The primary analysis was conducted…” later in the paragraph?

q13 AU: 80.3% (388/483) correct?

q14 AU: Text about soft-tissue reconstruction deleted for concision (these data are cited at end of current paragraph); OK?

q15 AU: (A) Insertion of %s in this sentence OK? (B) Please harmonize “single-stage or two-stage treatment of prosthetic joint infection” with the text of the relevant subheadings in Table 1.

q16 AU: Insertion of 18.3% correct?

q17 AU: Sentence OK?

q18 AU: Per the deputy editor, the change requested in your response to Q54 cannot be made, and the final sentence of the paragraph has been removed. The deputy editor has approved insertion of the following sentence if you believe it is needed (please advise): “However, although data from the current trial support the utility of local antibiotics in reducing the need for prolonged systemic antibiotics, a substantial amount of high-quality data is needed before local therapy is adopted as the primary approach in clinical practice.”

q19 AU: Current and remaining sentences in paragraph OK?

q20 AU: “secondarily on long-duration systemic therapy” OK?

q21 AU: Per the deputy editor, we have not inserted the requested sentences at the end of the main text per our policy regarding speculation and commentary about the manuscript (such remarks are for neutral third parties to make). Please acknowledge.

q22 AU: Changes to “Recruitment center” and “Débridement for OM or FRI with device removal or exchange” OK?

q23 AU: Change OK to harmonize with text?

q24 AU: Change OK to harmonize with text?

q25 AU: Appropriate to delete this row and change subheading of third row above this to “Two-stage revision for prosthetic joint infection”?

q26 AU: My apology for the lack of clarity. Please provide the range of possible scores on the CCI (perhaps 0 to 37?).
