# Supplementary material for: Short or Long Antibiotic Regimens in Orthopedics
Source: N Engl J Med. Author manuscript; Available in PMC 2026 Oct 7. (PMC7619510; doi:10.1056/NEJMoa2506756)
Supplement: Supplementary file 1 [file EMS217992-supplement-supplement.pdf]

Supplementary Appendix

Table of Contents

List of SOLARIO Investigators……………………..……………………………1

Acknowledgements……………………………………………………………2

Introduction…………………………………………………………………..3

Background to the Patient Population……………………………………….4

Inclusion and Exclusion Criteria…………………...…………………………4

Endpoint Committee Definitions of Failure…………………………………6

Post-randomisation participant non-eligibility………………………….…8

Choice of Antimicrobial and Carrier Material………………………………..8

Supplementary Analyses………………..…………………………………9

Supplementary Figures referenced in main text.…………………………12

Supplementary Figures referenced in appendix…………………………..15

Supplementary Tables…………………………………………………………..21

References……………………………………………………………………27

List of SOLARIO Investigators

Martin McNally[1], Maria Dudareva[1,2], Michelle Kumin[2], Werner Vach[3], Claire Scarborough[2], Matthew Cicero[4], Ricardo Sousa[5], Jamie Ferguson[1], David Stubbs[1], Bridget Atkins[1], Alex Ramsden[1], Bernadette Young[1,2], Andre Carvalho[5], Adrian Kendal[1], Abtin Alvand[1], Antony Palmer[1], Con Loizou[1], Ben Kendrick[1], Adrian Taylor[1], Amiral Islam[6], Michael Riste[6], Anand Pillai[6], Sunil Sharma[7], Jonathan Folbe[8], Neal Jacobs[9], Simon Ellis[10], Aaron Ng[11], Daniel Perez-Prieto[12], Irene Sigmund[13], Reinhard Windhager[13], Alexis Iliadis[14], Andrew Bing[15], Henry Wynn Jones[16], John Williams[17], Ben Ollivere[18], Volker Alt[19], Simon Warren[20], Neil Jenkins[21], Andreas Leithner[22], Harriet Hughes[23], Steve Jones[24], Martin Williams[25], Matthew Scarborough[1,2].

1) Bone Infection Unit, Nuffield Orthopaedic Centre, Oxford university Hospitals, UK
2) Nuffield Department of Medicine, University of Oxford, UK
3) Basel Academy for Quality and Research in Medicine, Switzerland
4) Great Western Hospitals Foundation NHS Trust, UK
5) Hospital University Centre of Porto, Portugal
6) Manchester University NHS Foundation Trust, UK
7) Brighton and Sussex University Hospitals NHS Trust, UK
8) Royal Liverpool and Broadgreen University Hospitals NHS Trust, UK
9) Salisbury NHS Foundation Trust, UK
10) Northumbria Healthcare NHS Foundation Trust, UK

11) Mid-Yorkshire Hospitals NHS Trust, UK
12) Institut Hospital del Mar d'Investigacions Mèdiques, Spain
13) Medizinische Universität Wien, Austria
14) Barts Health NHS Trust, UK
15) The Robert Jones and Agnes Hunt Orthopaedic Hospital NHS Foundation Trust, UK
16) Wrightington, Wigan and Leigh NHS Foundation Trust, UK
17) South Tees Hospitals NHS Foundation Trust, UK
18) Nottingham Biomedical Research Centre, School of Medicine, University of Nottingham, UK
19) University Hospital Regensburg, Germany
20) Royal National Orthopaedic Hospital NHS Trust, UK
21) University Hospitals Birmingham NHS Trust, UK
22) Medizinische Universität Graz, Austria
23) University Hospital of Wales, UK
24) Cardiff & Vale University Health Board, UK
25) University Hospitals Bristol NHS Foundation Trust

Data Safety and Monitoring Committee
Neil French
Graham Cooke
Ly-Mee Yu

Blinded Endpoint Committee
Harriet Hughes
Steve Jones
Martin Williams

Patient Representatives
Jennifer Bostock
Helen Campbell
Heather Gibbs
Susan Hinde
Margaret Ogden
Deb Smith
David South

Acknowledgements

We thank the patients who kindly consented to be part of the trial and the staff of the Oxford Bone Infection Unit. We are very grateful to Shrista Lama, Shauna Masters, Florian Frank, Mark Farrar, Charline Roslee, Edward Gardner, Ross Muir, Bambos Charalambous, Dunisha Samarasinghe, Graham Sweeney, Amanada Wood, Fiona

Fitzgerald, Valerie Parkinson, Athanasia Filippa, Jill Woodford, Helen Langton, Tracey Smith, Gail Waddell, Theresa Garratt, Dee Mullan, Julie Newman, Jane Gaylard, Denise Skinner, Charlotte Bentley, Alphonsa Biju, Jossy Kayappurathu, Bini George, Sarah Diment, Sarah Buckley, Victoria Cunliffe, Shamina Hankinson, Rachel Shranz, Charlotte Humphrey, Kim Dearnley, Rebecca Wood, Adele Szekeres, Sandra Hacker, Maggie Breisach, Tanja Dengler, Jennifer Bostock, Mohamed Haniff and Fraser Old for their help with recruitment and patient management during the trial.

Design and Conduct of the Study

The study was principally designed by M McNally, M Scarborough and M Dudareva. The statistical analysis was designed by W Vach, M McNally and M Dudareva. Data was gathered in each trial centre by the co-workers acknowledged above under the supervision of investigators in each site. Data was principally analyzed by M McNally, W Vach and M Dudareva, who vouch for the data. The original draft of the paper was written by M McNally and M Dudareva with contributions from all co-authors in subsequent versions. No payment was received for writing and no AI was used in the production of the text or any part of the work.

There are no agreements concerning confidentiality of the data, other than the normal protection of patient information to maintain anonymity.

Introduction

Musculoskeletal conditions account for a large burden of disease around the world[1]. Treatment of these conditions has improved dramatically in recent decades but bone and joint infections, particularly after joint replacement or fracture, continue to produce substantial disability[2,3]. There are around 2 million new fracture-related infections (FRIs) each year, the majority affecting patients in low or middle income countries[4,5]. Prosthetic joint infection (PJI) is now the most common cause for revision of arthroplasties. It is often required in older patients with complex comorbidities and is technically demanding and resource intensive[6-8].

Recent animal studies have suggested that local antimicrobials alone may be sufficient to eradicate bone infection, without the need for prolonged systemic antibiotics[9], but this has not been tested in humans.

Most studies of the use of local antibiotics in bone or joint infection have not included any change in the standard of care with regard to the duration of systemic antibiotic therapy. It has therefore been difficult to define the relative contribution of systemic or local therapy to the outcome of treatment. There is no high quality evidence to compare the effects of local and systemic therapy. The recent trend to add local antibiotic implantation to treatment regimes, which already include long systemic antibiotic courses, increases the total consumption of antimicrobials and goes against recommendations for improved antimicrobial stewardship[10].
This trial did not compare local versus systemic antibiotic therapy, but it shows that, where local antibiotics are included, there is no advantage of adding a prolonged (≥ 7 days) course of systemic antibiotic therapy.

Background Information on the Patient Population

The Global Burden of Disease Project [1,10] does not specifically record MSK infection, but estimate that the prevalence of MSK conditions overall is greater in higher income countries: "High-income countries are the most affected in terms of number of people – 441 million – followed by countries in the WHO Western Pacific Region with 427 million and South-East Asia Region with 369 million. Bone and joint infections related to prosthetic replacement and internal fixation of fractures will be more prevalent in high-income countries, due the wider availability of these treatments [3-5], while childhood haematogenous osteomyelitis may be more prevalent in low and middle-income countries. Musculoskeletal conditions are also the biggest contributor to years lived with disability (YLDs) worldwide with approximately 149 million YLDs, accounting for 17% of all YLDs worldwide.

Table S1 describes the specific issues of representativeness of the SOLARIO trial.

Inclusion criteria

The participant must meet ALL of the following criteria:

1) Provision of informed consent
2) Aged 18 or over
3) Presenting with an orthopaedic infection, characterised by one or more of the following:

- Localised pain OR
- Localised erythema OR
- Temperature ≥ 38° C
- A discharging wound or sinus

4) Undergoing definitive (curative) surgical treatment for the infection
    - Surgery must be performed as the definitive procedure to eradicate the infection. The intention should not be to reduce the bioburden and then suppress with long-term antibiotics until a further stage. Similarly, serial washout/debridement procedures, abscess drainage or minimal sampling or partial excisions were excluded.
5) Locally administered antibiotics in an HTA-approved product or in a carrier CE marked for antibiotic delivery
6) Has received ≤ 7 days systemic antimicrobial therapy since surgery
7) Would ordinarily be managed with a prolonged course (≥ 4 weeks) of systemic antibiotic therapy
8) Specimens for microbiological analysis taken at index surgery.

All patients had deep tissue sampling, excision of dead bone, removal of infected metalware/prostheses, implantation of local antibiotic in a licenced carrier and skin closure. In patients requiring stabilization after debridement, the surgeon could choose internal or external fixation for fracture-related infections or osteomyelitis. In prosthetic joint infections, antibiotic loaded spacers, with or without additional pellet antibiotics were used. Reimplantation could be in a single stage surgery or over two stages.

We included patients with:

1) Prosthetic joint infection receiving single stage revision, two-stage revision or excision arthroplasty.
2) Osteomyelitis with debridement in the absence of implanted metalware
3) Osteomyelitis with debridement and removal <u>or</u> exchange of implanted metalware
4) Fracture-related infection with debridement and removal <u>or</u> exchange of metalware
5) Diabetic foot osteomyelitis with debridement
6) Complex native septic arthritis with debridement of adjacent bone (usually excision arthroplasty)

Debridement of dead bone was mandatory and could be localized or segmental. Local antimicrobial delivery could be achieved with implantation of absorbable or non-absorbable carriers, as beads, paste, spacers, rods or combinations of these.

PJI treated by two-stage revision was included. The first stage was the index procedure and included the implantation of local antibiotics and skin closure. If infection was present at stage two (diagnosed by definition criteria and microbiological culture), this was referred to the blinded end-point committee for evaluation as a potential failure.

Infection was confirmed after surgery based on the presence of clinical features (as above), positive microbiological cultures and/or positive histological criteria for infection as recommended in definitions for each infection type [11-13] (Table 1).

Exclusion criteria

Surgical exclusions

1) The index surgery was not a definitive procedure with the aim of eradicating infection
    - Primary closure has not been achieved
    - Re-look surgery planned
2) The index operation involved implant retention (e.g. DAIR)
    - This exclusion affected both prosthetic joint infections treated with DAIR and fracture-related infections with any retained metalware.

Microbiological exclusions

1) Any micro-organisms from the operative specimens that are fully resistant to the local antibiotic(s) implanted at the site of infection

Medical exclusions

1) Other infection necessitating additional systemic antibiotic treatment beyond 7 days after surgery, such as *Staphylococcus aureus* bacteraemia, psoas abscess or bacterial endocarditis.
2) Patient in another clinical trial involving an investigational medicinal product (IMP) related to infection.

End-point committee definitions for definite, probable and possible treatment failure

Definite treatment failure was defined by one or more of the following:

1) The isolation by culture of micro-organisms from 2 or more samples of bone/peri-prosthetic tissue from the site of incident infection at re-operation, where the micro-organisms are phenotypically indistinguishable*.

2) A pathogenic organism (e.g. *Staphylococcus aureus* but not *Staphylococcus epidermidis*) on a single, closed, biopsy or aspirate from the site of incident infection.

3) Histology diagnostic of infection from bone/peri-prosthetic tissue obtained at re-operation at the site of incident infection.

4) Formation of a sinus tract arising from the bone/prosthesis/orthopaedic device identified at clinical review or re-operation.

5) Recurrence of frank pus adjacent to bone/prosthesis/orthopaedic device identified at re-operation or aspiration

6) Elevated synovial fluid White Cell Count or neutrophil percentage, or ++ change on leucocyte esterase strip, from prosthetic joint site affected by incident infection, at re-operation or aspiration.

7) Death resulting from orthopaedic infection at the incident anatomic site.

* indistinguishable refers to the results of routine laboratory testing, including bacterial genus/species and the results of routine antibiotic susceptibility testing. We did not require any additional bacterial typing in the laboratory beyond local routine practice.

Probable treatment failure was defined when none of the criteria for definite infection had been fulfilled AND any one of the following were met:

a) The development of a discharging wound at the site of the bone/prosthetic joint/orthopaedic device at clinical review OR

b) Pus close to but not adjacent to bone/prosthetic joint/orthopaedic device at reoperation OR

c) The presence of peri-prosthetic necrotic bone on operative histology.

d) Amputation at the site of initial orthopaedic infection.

e) Erythema, warmth and localized pain at the site of previously treated infection on clinical review OR

f) Radiological or operative findings of periosteal changes suggesting osteomyelitis or prosthetic joint infection OR

g) Rapid loosening of a joint prosthesis/orthopaedic device (i.e. leading to localized pain in less than 3 months since implantation), in the absence of a mechanical explanation for rapid loosening, on radiological, clinical or operative examination

Possible treatment failure was defined where microbiological sampling had been undertaken with negative results AND other criteria for definite infection were not fulfilled AND in addition one or more of the criteria listed a) to g) above was met.

Where the blinded endpoint committee members differed in their initial categorization of a participant, consensus was achieved by discussion or, if necessary, by majority opinion. The endpoint committee, if necessary, took into account additional factors not listed above in the categorization of endpoints as definite, probable or possible.

Post-randomisation participant non-eligibility

Three participants withdrew their consent for follow-up, including the use of their data in trial analysis, shortly after enrolment. Two further participants withdrew their consent for follow-up, while allowing their data to be used in analyses.

Twenty-two participants were excluded due to non-concordance with eligibility criteria, or withdrawal from follow-up (2 participants). For 7 participants, this was due to incomplete assessment at the time of randomisation: 1 participant had retained infected metalwork at the site of infection (DAIR), 4 participants did not undergo wound closure at the completion of the index operation as planned, 1 participant did not have local antimicrobials implanted during surgery, and one participant was randomised more than 7 days after surgery. For the other participants, additional exclusion criteria were identified after randomisation: alternative diagnoses to infection for two participants, and micro-organisms fully resistant to implanted local antibiotics for 11 participants (Figure 1, CONSORT Diagram).

Choice of Local Antimicrobial and Carrier Material

The choice of implanted carrier material and the included antibiotic was at the discretion of the treating surgeon. This trial was not a study of any specific carrier material or group of materials but rather the concept of delivering antimicrobial therapy locally from a licenced material providing a high concentration of the included antibiotic over at least a four week period. All carriers in the study have been licenced after appropriate elution and safety studies.

In general, PJI cases were treated with PMMA-based carriers and FRI/OM cases had bioabsorbable carriers but there was some overlap in this principle.

In many modern licenced local carriers, the antibiotic content is predetermined by the manufacturer (e.g. Cerament G with 17.5mg of Gentamicin per ml of calcium sulphate/hydroxyapatite carrier). However, several carriers (predominantly PMMA and Stimulan) allow surgeon mixing of antibiotics. This was performed in 71 long arm cases and 63 short arm cases. Table S2 shows the breakdown of carriers by treatment arm.

Gentamicin was the most frequently used local antibiotic with a mean implanted dose of 246mg in absorbable carriers and 2280mg in PMMA-based carriers. Other local antibiotics included tobramycin and vancomycin. Table S3 describes the included antibiotics and their doses (median and IQR).

Supplementary Analyses

Sensitivity analyses with respect to the handling of patients lost to follow up

There were 34/241 failures in the long group and 26/234 in the short group, among eligible patients. If all 8 patients lost to follow-up in the short group are regarded as failures and all 3 patients in the long group are regarded as successes, this would produce a total failure rate of 34/234 (14.5%) in the short group and 34/241 (14.1%) in the long group.

The analyses in the manuscript are based on the assumption that patients lost to follow up do not hide any failure. The robustness of the results with respect to this assumption are investigated in sensitivity analyses presented here. Figures S4 to S6.

The sensitivity analyses are based on different assumptions about the true percentages of failures among the 8 patients lost to follow-up in the SHORT arm and the 3 patients lost to follow up in the LONG arm. The percentages in the SHORT arm were varied from 0% to 100%, and the percentages in the LONG arm were assumed to be equal to those in the SHORT arm, reduced by 50%, or they were set to 0%. (The assumption of a percentage of 0% in the long arm and of 100% in the short arm represent the worst-case scenario.)

For each pair of percentages considered, the corresponding number of failures among all eligible patients was determined, rounded to the next integer, and the main analysis was performed as described in the manuscript.
The three figures present the results with respect to the primary outcome and the two alternative definitions of the primary outcome. The red line indicates the non-inferiority margin.
With respect to the primary outcome “definite failure”, only for the most extreme pair of percentages did the risk difference become positive, and even in this worst case the confidence interval does not include the non-inferiority margin.

Assessment of the proportional hazard assumption with regard to mortality

The standard graphical checks of the proportional hazard assumption were performed and are illustrated in Figure S7.

In summary, a violation of the proportional hazard assumption cannot be excluded. However, due to the limited number of events, there is no clear evidence for a violation. A formal test based on the slope of the Schoenfeld residuals yields a p-value of 0.096.

Taking into account that:

- survival is a secondary outcome
- the aim was to exclude a potential harm due to the reduced used of antibiotics and not to demonstrate a better survival in the SHORT arm than in the LONG arm
- alternatives to using a hazard ratio to describe survival differences between two groups are still not well established,

it can be justified to use the hazard ratio to quantify the difference in survival and to base statistical inference on a hazard ratio, even if there can be some doubts about the validity of the proportional hazard assumption.

Center-specific outcomes for definite failure

Figure S8 provides information about the number of patients and the rate of definite failure at each center.

Figure S9 provides information about the number of patients and the number of definite treatment failures in each arm at each center. In addition, the estimated risk differences are shown. In only four centers, the estimated risk difference was above the non-inferiority margin, but these centers contributed only 7 failures, in total, to the overall failure rates.

Subgroup analyses for age and the Charlson comorbidity index.

In order to avoid dichotomization of these two variables, they were reported based on a regression model analysis including interaction terms. The model included as covariates the treatment, the variables of interest as continuous covariate, and the interaction between treatment and the variable. Such a model can also be interpreted as modelling the treatment effect as a linear function of the continuous covariate and hence allows directly to determine the treatment effect at specified covariate values [14].

The covariate values chosen were the lower and upper 10th percentiles of the covariate in the population of eligible patients.

The regression model used was a generalized linear model with identity link and assumed variance according to a Bernoulli distribution. This model allows interpretation of treatment effects directly as risk differences (Figure 3).

Supplementary Figures

Supplementary Figures Referenced in the Main Manuscript

Supplementary Figure S1 **Frequency of definite, probable and possible treatment failures in the three analysis populations**

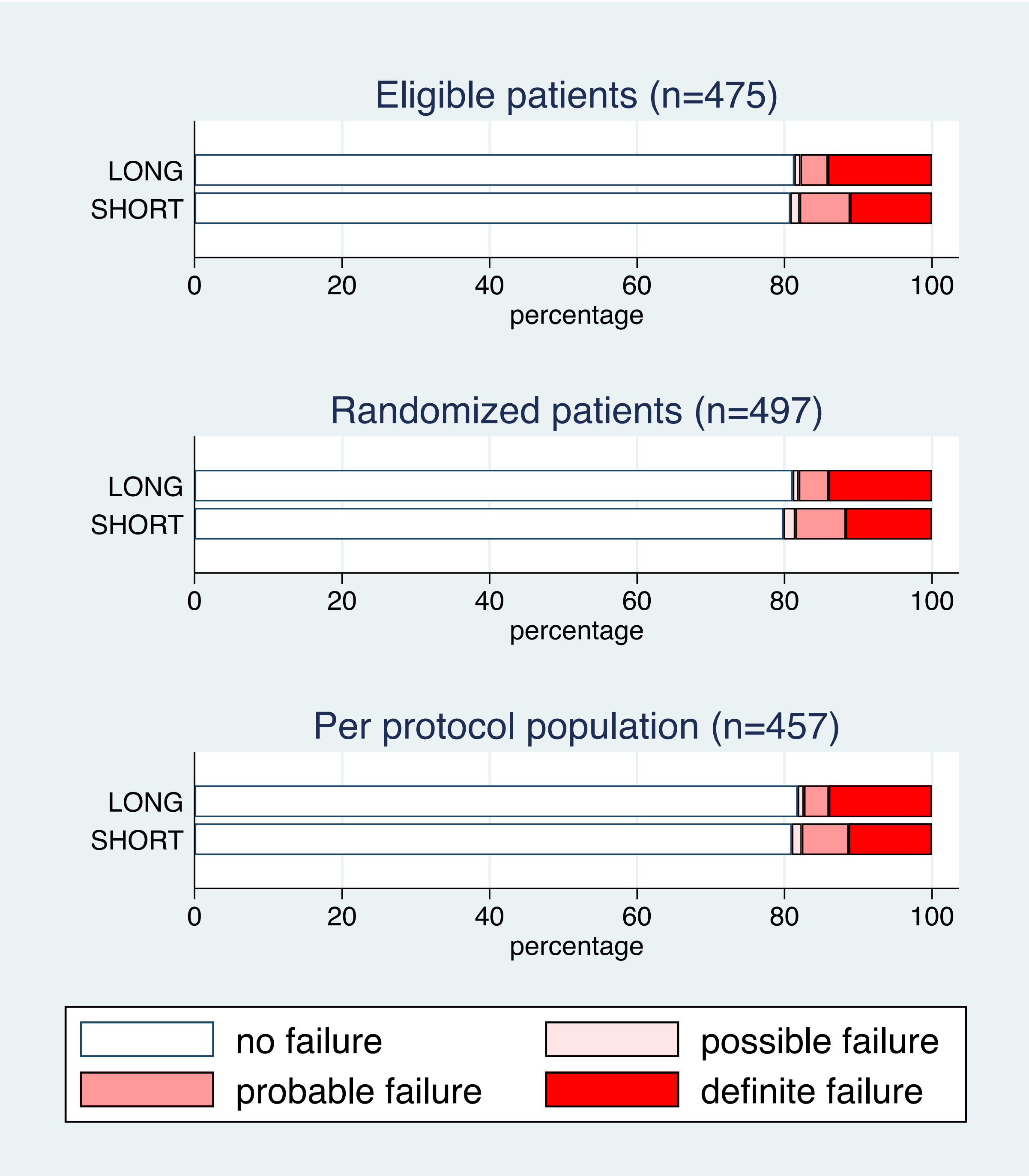

Supplementary Figure S2 **Treatment arm-specific distributions of length of hospital stay and overall survival***

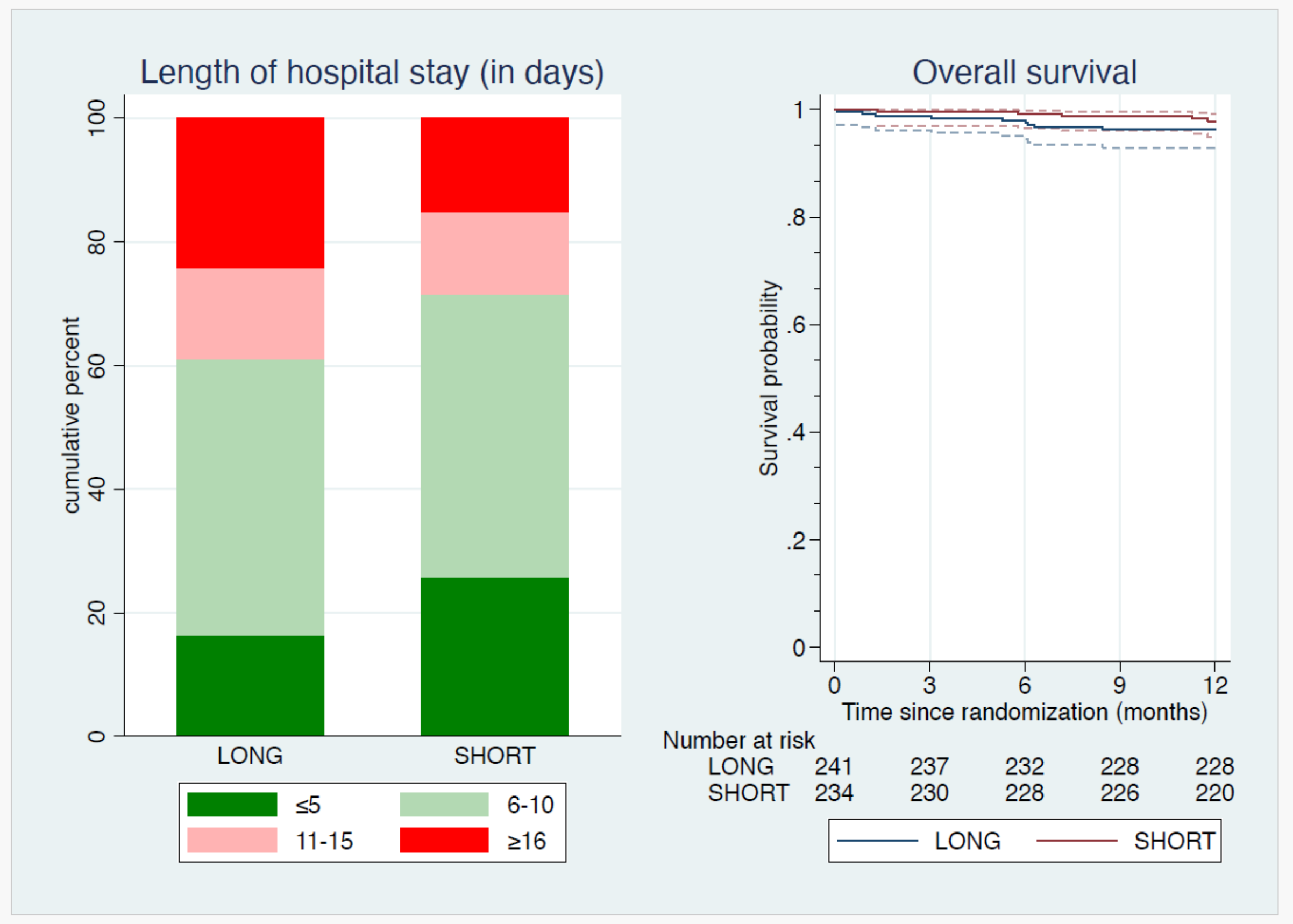

*Pointwise 95% confidence limits are shown as dashed bands

Supplementary Figure S3 **The incidence of symptoms reported at 6 weeks and 3-6 months which may be related to antibiotic therapy**

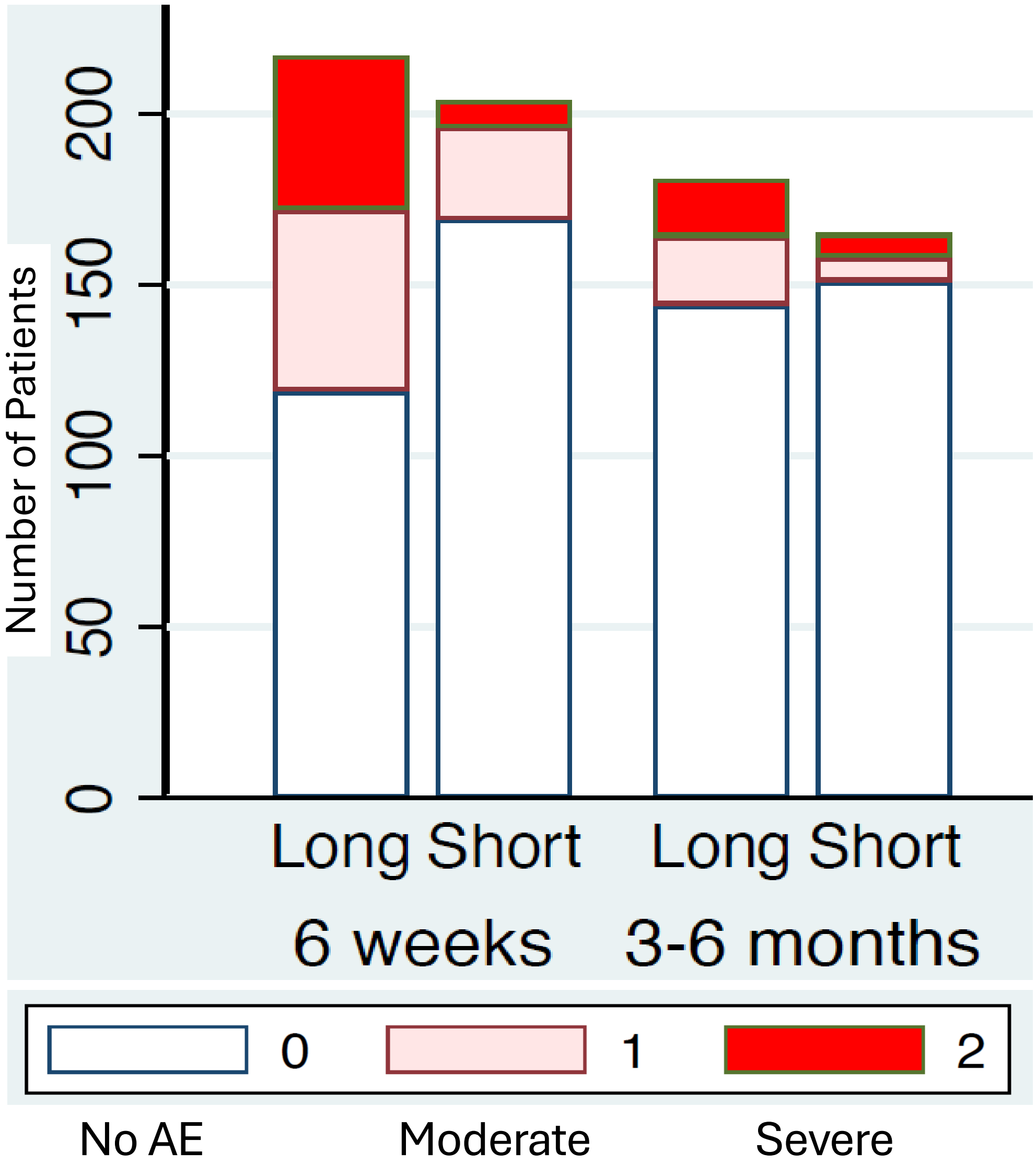

Supplementary Figures Referenced in the Appendix

Supplementary Figure S4 **Sensitivity analyses for Definite failure only**

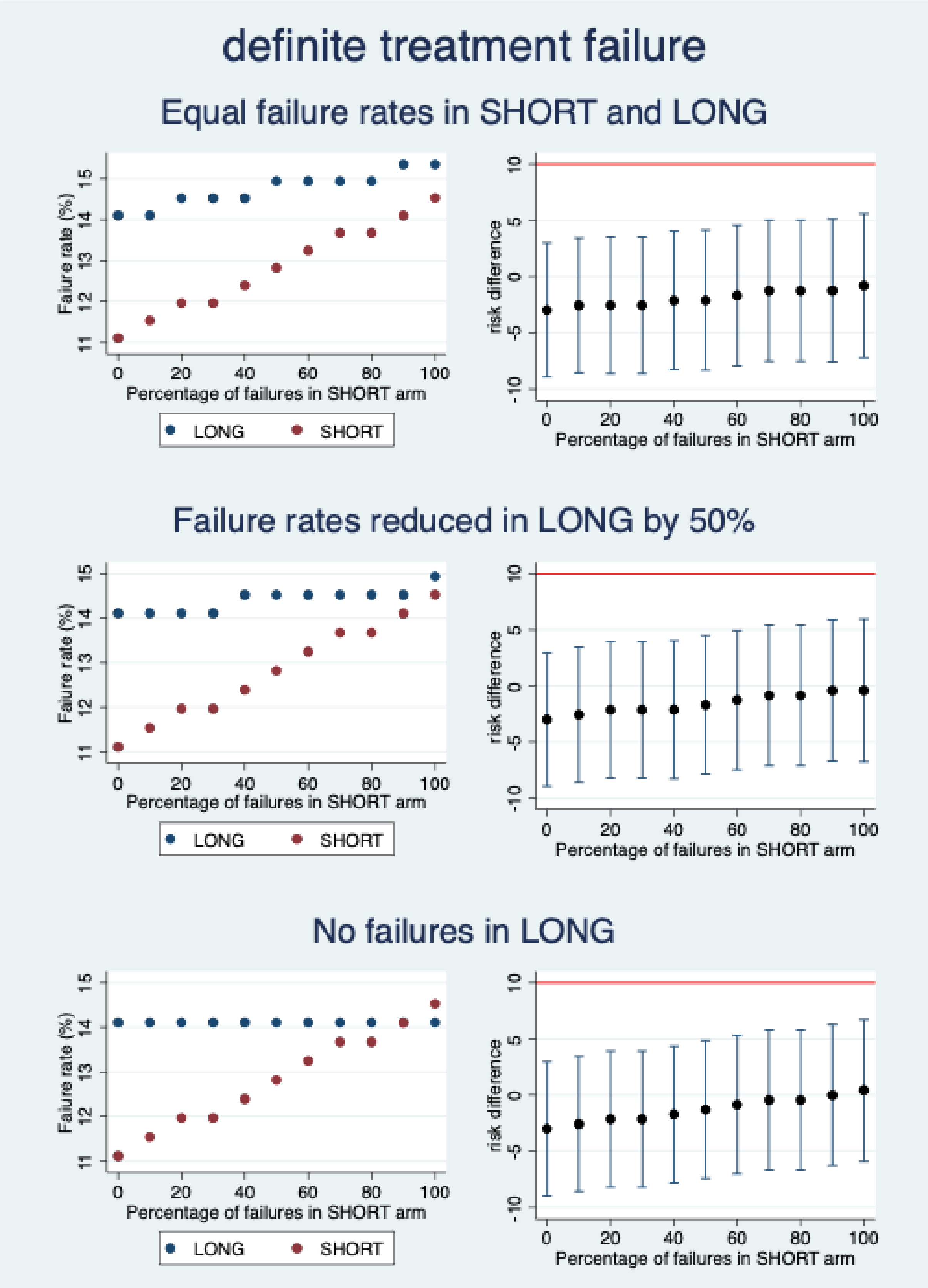

Supplementary Figure S5 **Sensitivity analyses for Definite or Probable failure**

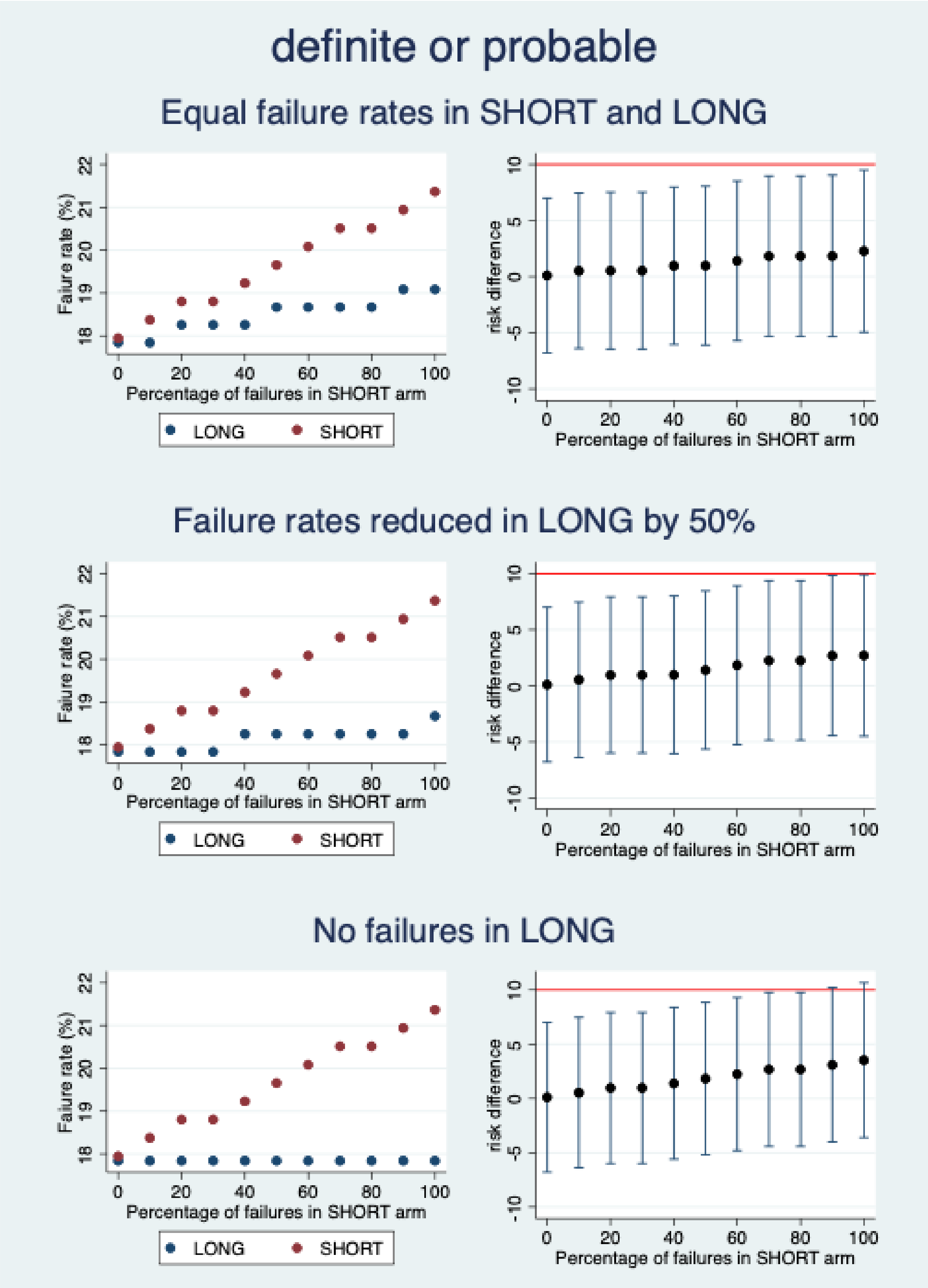

Supplementary Figure S6 **Sensitivity analyses for Definite or Probable or Possible failure**

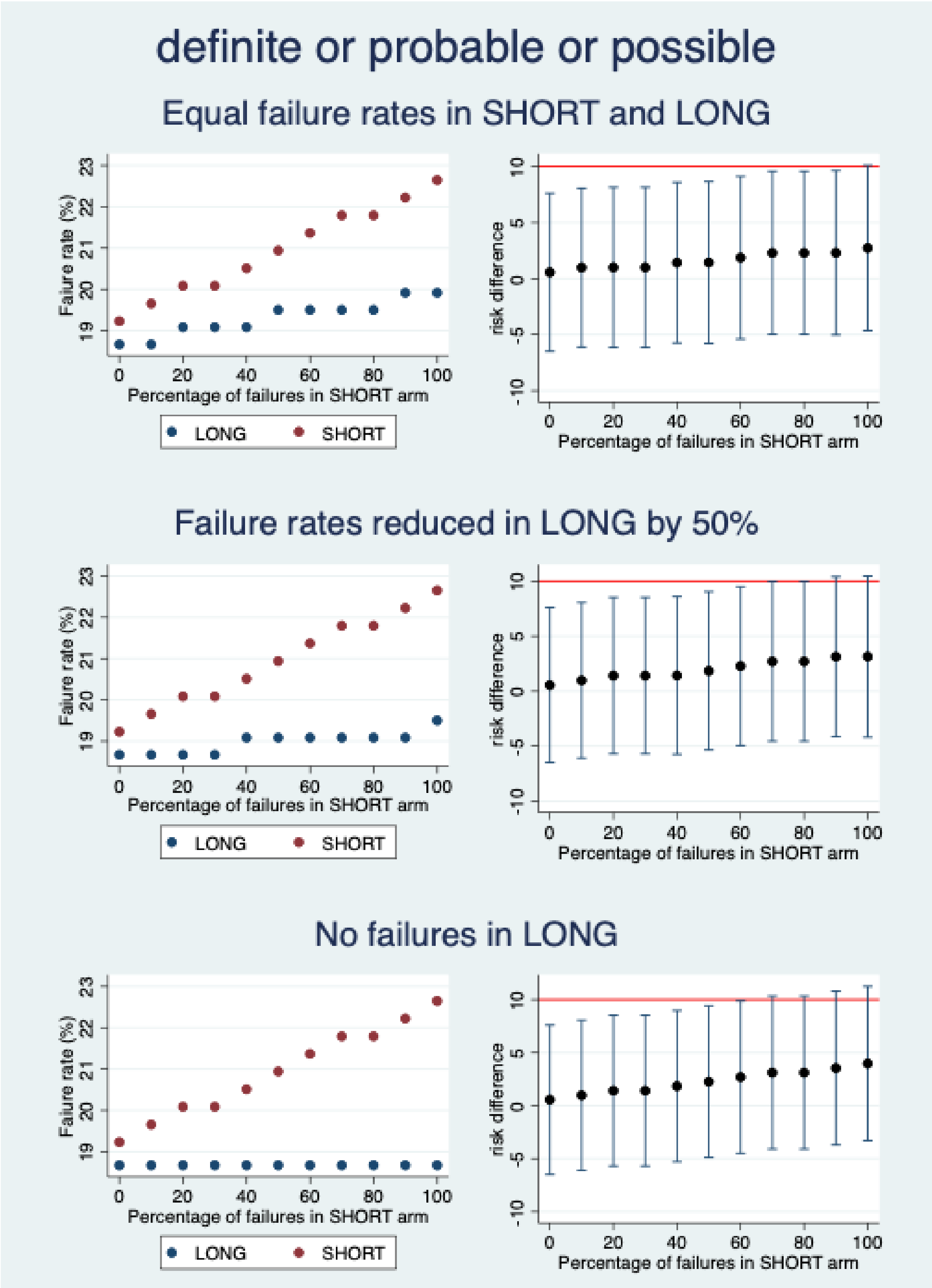

Supplementary Figure S7 **Assessment of the proportional hazard assumption with regard to mortality**

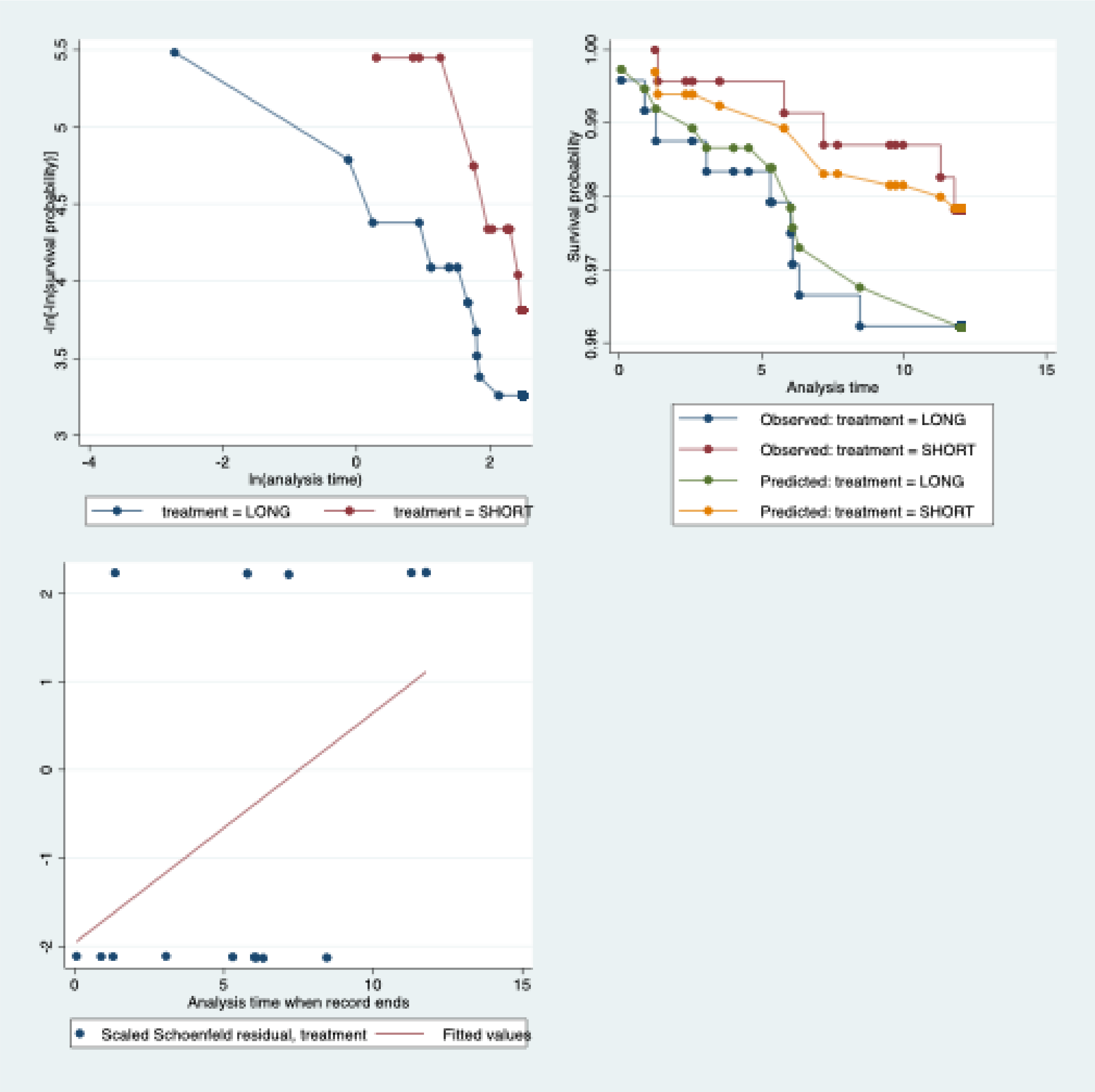

The log-log plot indicates some deviation from two parallel lines to be expected under the proportional hazard assumption. The predicted values from the Cox model slightly deviate from the observed values, indicating a slight underestimation of the treatment effect. The slope in the plot of the scaled Schoenfeld residuals indicates a slope different from 0.

Supplementary Figure S8 **The Distribution of Recruitment and Failure Rates at each Center**

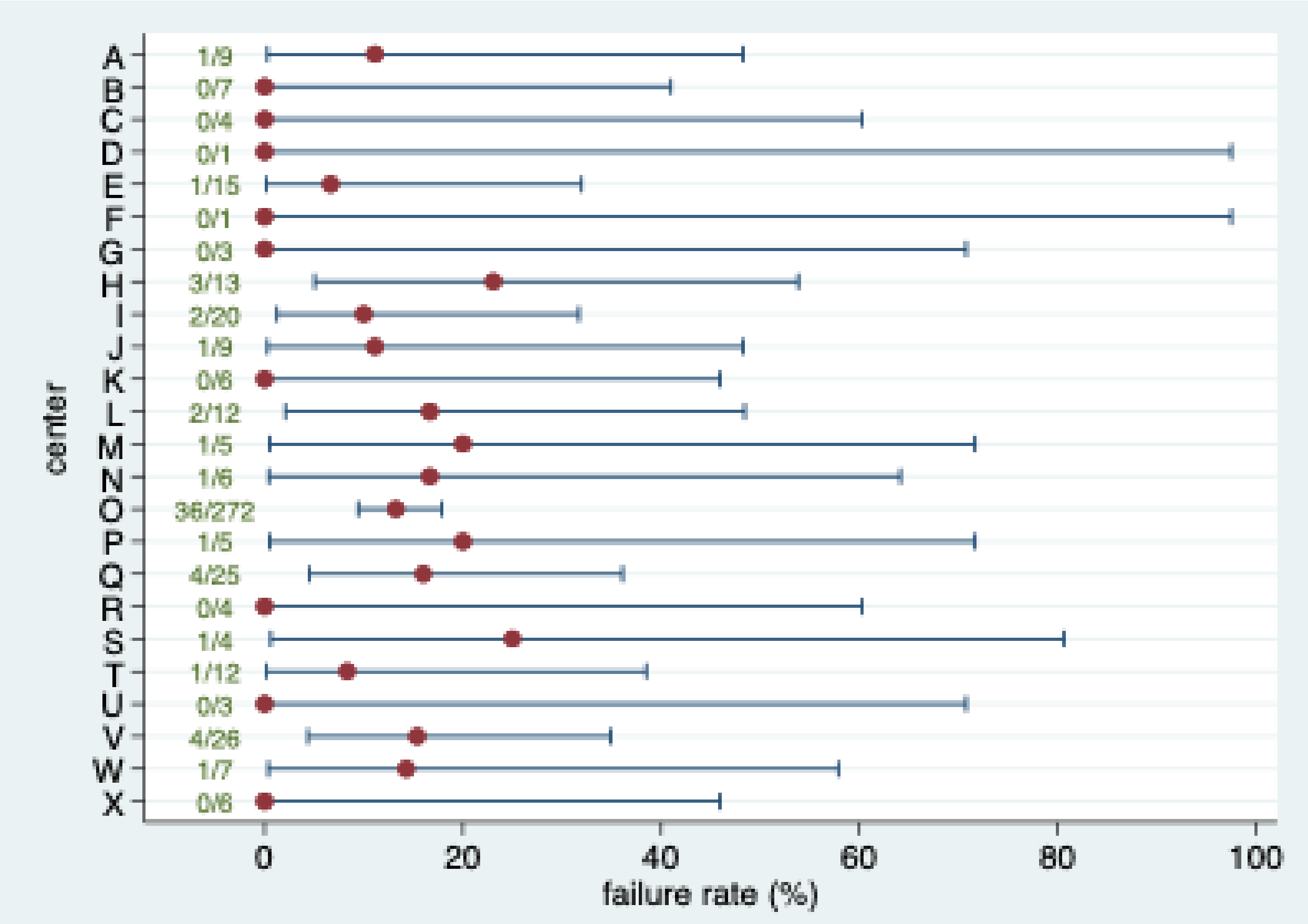

The number K of definite treatment failures, the number N of treated patients (represented as K/N in green) and the observed failure rate with 95% confidence intervals, stratified by center.

Supplementary Figure S9 **The Distribution of Definite Treatment Failures by Center and Treatment Arm**

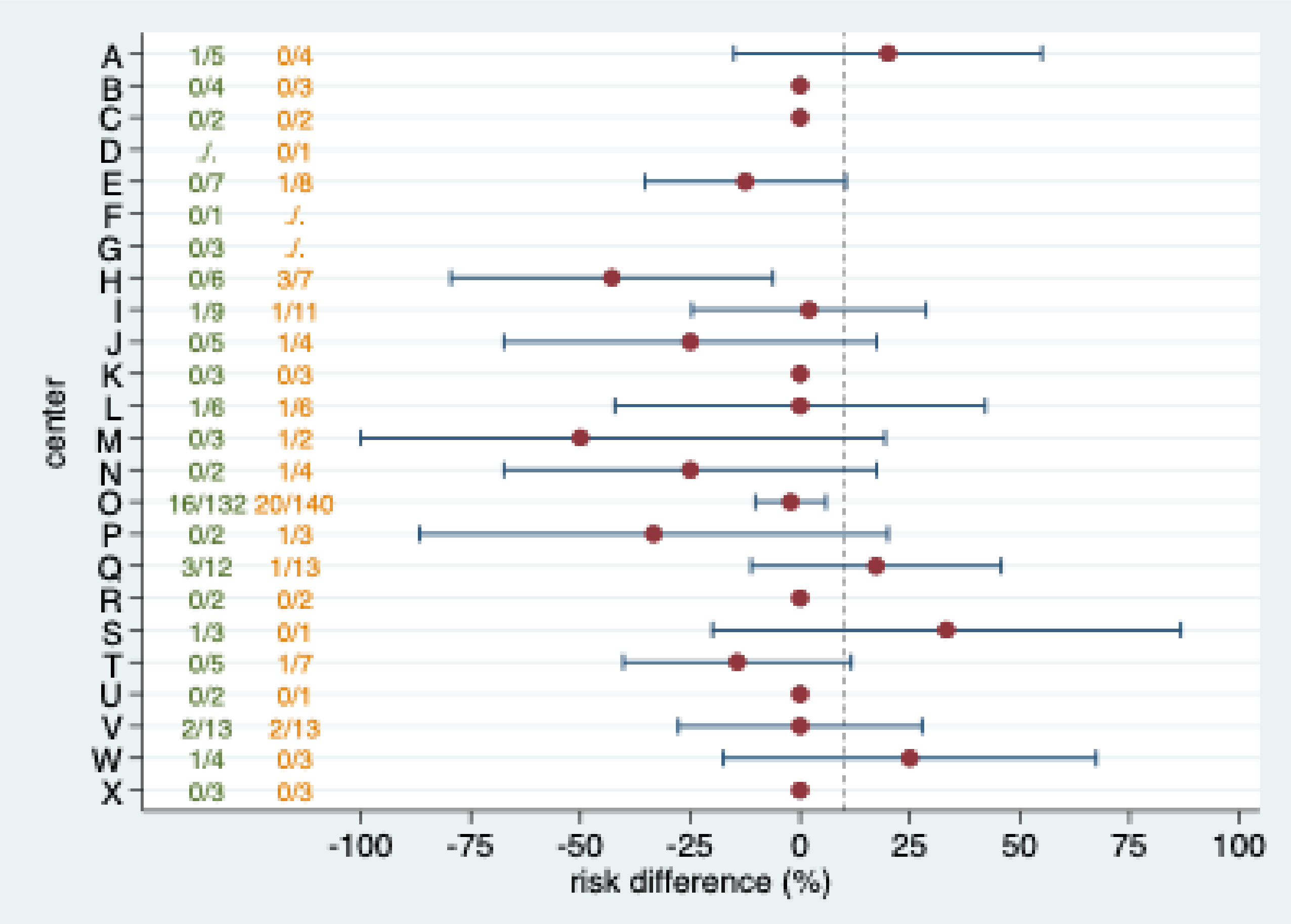

The number K of definite treatment failures and the number N of treated patients (represented as K/N) in the SHORT arm (in green) and in the LONG arm, (in orange) for each center. In addition, the estimated risk difference is shown together with a 95% confidence interval. For centers only contributing patients in one treatment arm, no estimate of the risk difference could be computed. For centers with no treatment failure, no confidence interval was computed. The vertical dashed line indicates the noninferiority margin.

Supplementary Tables

Supplementary Table S1 Information of the Patient Population and Representativeness of the SOLARIO Trial

Category	Description
**Age**	Orthopaedic infection has a bimodal age distribution which peaks during childhood, when infection is mainly haematogenous in origin, and in later adulthood, when the prevalence of comorbidities, surgically managed fractures, diabetic foot infection and prosthetic joint infection increases.<br>SOLARIO recruited all types of infection from the adult peak of orthopaedic infection. Paediatric infection was excluded.
**Gender**	Osteomyelitis, fracture-related infection and prosthetic joint infections are more common in males than in females. This is likely to be due to greater exposure to trauma, peripheral vascular disease and diabetes mellitus.<br>SOLARIO recruitment confirmed this predominance of males (71% vs 29% female)
**Race and ethnicity**	There is little data available comparing the incidence, severity or outcomes of musculoskeletal infection between ethnic groups. Studies of the microbiology in Europe and Africa have shown very limited differences in the range of pathogens cultured, with *Staphylococcus aureus* predominating in all regions.<br>We did not specifically collect data on race or ethnic origin. However, we have more general data from our centers which showed that during the period of recruitment, about 80% of our patients were White European, 1.5% were Black British, Black Caribbean or Black African and 3% were South or East Asian. All of our centers were tertiary referral centres and received patients of all ethnicity and race without restriction.
**Geography**	Although the trial was performed in sites in Europe, there is no clear reason to suggest that the findings would not be applicable in other high-income areas, or in ethnic minority populations or low and middle income countries (LMICs) in which the incidence of bone and joint infection is higher. There is no evidence that the duration of systemic antimicrobial therapy should be different in other geographic areas.
**Representativeness**	The current trial was restricted to adults over the age of 18 years. Whilst we cannot presume that the results can be extrapolated to paediatric practice, it is biologically unlikely that the efficacy of local antibiotics would be limited in younger patients.<br>There were no exclusion criteria based directly upon ethnicity, and inclusivity was facilitated by translation of the patient information sheet and consent form into multiple languages, and through the use of professional interpreting services.<br>Whilst the trial did not recruit in LMICs, the strategy of using local antibiotics may have particular advantages in such settings on account of the cost of systemic therapy, consequent concerns over adherence, and the risks associated with suboptimal antimicrobial stewardship.

Supplementary Table S2 **The Distribution of Antibiotic Carriers by Treatment Arm**

	**Long Antibiotics Arm** n=249 n (%)	**Short Antibiotics Arm** n=248 n (%)
**Carrier Type**		
Bioabsorbable	216 (87)	207 (83)
Polymethylmethacrylate Cement (PMMA)	53 (21)	46 (19)
Both carriers types	26 (10)	11 (4.4)
**Carrier Product**		
Cerament G	137 (55)	143 (58)
Cerament V	78 (31)	68 (27)
Herafill G	27 (11)	21 (8.5)
Osteoset T	32 (13)	30 (12)
Stimulan* with antibiotics	27 (11)	18 (7.3)
PMMA with antibiotics	33 (13)	27 (11)
Preformed PMMA Spacer or Beads	7 (2.8)	4 (1.6)

*Stimulan with added antibiotics was not used alone but was most often added to the joint cavity in patients having treatment of PJI with PMMA cemented arthroplasty.

Supplementary Table S3 **The Distribution of the most common Antimicrobials by Treatment Arm**

	**Long Antibiotics Arm** n=249	**Short Antibiotics Arm** n=248
Number of Patients Treated		
Gentamicin	205	196
Vancomycin	160	144
Tobramycin	34	35
Clindamycin	3	4
Dose of Antibiotic*		
Gentamicin dose	0.18 (0.09, 0.50)	0.18 (0.09, 1.00)

Vancomycin dose	1.00 (0.66, 2.00)	1.00 (0.66, 1.32)
Tobramycin	1.20 (0.40, 1.60)	1.20 (0.80, 1.35)
Clindamycin	2.00 (1.50, 2.50)	1.50 (1.00, 2.75)

*All doses are expressed as the median in grams with interquartile range.

Supplementary Table S4 **Distribution of Mode of Failure within the Composite Definite Failure Endpoint by Treatment Arm**

Long Antibiotics Arm (n=241)								
	**Mode of Failure**							**Frequency**
	1	*2*	*3*	*4*	*5*	*6*	*7*	
	N*	N	N	Y	N	N	N	14
	Y#	N	N	Y	Y	N	N	4
	Y	N	Y	Y	N	N	N	3
	N	Y	N	N	Y	N	N	2
	N	Y	N	N	N	N	N	2
	Y	Y	Y	Y	Y	N	N	2
	N	N	Y	N	N	N	N	1
	Y	Y	N	N	Y	N	N	1
	Y	N	N	Y	N	N	N	1
	Y	Y	Y	N	Y	N	Y	1
	N	N	N	Y	Y	N	N	1
	N	Y	N	Y	Y	N	N	1
	Y	N	N	N	N	N	N	1
								Total 34

Short Antibiotics Arm (n=234)								
	**Mode of Failure**							**Frequency**
	1	*2*	*3*	*4*	*5*	*6*	*7*	
	Y	N	N	Y	N	N	N	6
	N	N	N	Y	N	N	N	5
	Y	N	N	N	N	N	N	3
	N	Y	N	N	Y	N	N	2
	N	N	N	Y	Y	N	N	1
	Y	N	Y	N	N	N	N	1
	Y	N	N	Y	Y	N	N	1
	Y	Y	Y	Y	N	N	N	1
	N	Y	N	N	N	N	N	1
	N	Y	N	Y	N	N	N	1
	N	N	N	N	Y	N	N	1
	N	N	N	N	N	N	Y	1
	Y	Y	N	N	N	N	N	1
	N	N	N	N	N	Y	N	1
								Total 26

1. *The isolation by culture of micro-organisms from 2 or more samples of bone/peri-prosthetic tissue from the site of incident infection at re-operation, where the micro-organisms are phenotypically indistinguishable*.*

2. *A pathogenic organism (e.g. Staphylococcus aureus but not Staphylococcus epidermidis) on a single, closed, biopsy or aspirate from the site of incident infection.*
3. *Histology diagnostic of infection from bone/peri-prosthetic tissue obtained at re-operation at the site of incident infection.*
4. *Formation of a sinus tract arising from the bone/prosthesis/orthopaedic device identified at clinical review or re-operation.*
5. *Recurrence of frank pus adjacent to bone/prosthesis/orthopaedic device identified at re-operation or aspiration*
6. *Elevated synovial fluid White Cell Count or neutrophil percentage, or ++ change on leucocyte esterase strip, from prosthetic joint site affected by incident infection, at re-operation or aspiration.*
7. *Death resulting from orthopaedic infection at the incident site.*

*N Mode of failure not present.

#Y Mode of failure present.

Supplementary Table S5 **Full Baseline Demographics of the Trial Participants**

**Baseline Characteristics of the Trial Participants** (n=497)*		
**Characteristic**	**Longer antibiotics (n=249)**	**Shorter antibiotics (n=248)**
Gender[1]		
Female	73 (29%)	71 (29%)
Male	175 (71%)	176 (71%)
Age at operation (years)	59 (IQR 49 to 69)	57 (IQR 44 to 68)
ASA grade		
1	42 (17%)	53 (22%)
2	119 (49%)	117 (49%)
3	79 (33%)	66 (27%)
4	2 (0.8%)	5 (2.1%)
Not known	7	7
Charlson Comorbidity Index[2]	2 (IQR 1 to 4)	2 (IQR 0 to 4)
BMI[3]	28.7 (25.0 to 33.3)	27.8 (23.8 to 32.1)
Diabetes mellitus	60 (25%)	54 (22%)
Unknown	7	5
Complications present[4]	26 (11%)	16 (6.7%)
Current smoker[5]	44 (18%)	43 (18%)
Peripheral vascular disease[6]	15 (6.3%)	12 (4.9%)
Immunosuppressive medication	23 (9.2%)	17 (6.9%)
Bone infection	193 (78%)	196 (79%)
Joint infection	66 (27%)	63 (25%)
Affected area		
Hip	28 (11%)	22 (8.9%)
Knee	26 (10%)	26 (10%)
Femur	39 (16%)	26 (10%)
Tibia/fibula	50 (20%)	71 (29%)
Ankle	15 (6%)	14 (5.6%)
Foot	46 (18%)	37 (15%)
Complex lower limb	11 (4.4%)	12 (4.8%)
Spine or pelvis	2 (0.8%)	1 (0.4%)
Shoulder	4 (1.6%)	9 (3.6%)
Humerus	12 (4.8%)	14 (5.6%)
Upper limb other	16 (6.4%)	15 (6.0%)
Unknown	0	1
Previous operations at same anatomical site[7]	212 (86%)	206 (85%)
Operation[8]		
Osteomyelitis/FRI debridement	87 (35%)	88 (35%)
Osteomyelitis/FRI with implant removal or exchange	75 (30%)	8% (34%)
Diabetic Foot Osteomyelitis debridement	31 (12%)	20 (8.1%)
Complex septic arthritis	9 (3.6%)	5 (2.0%)

Prosthetic joint removal or first stage revision	24 (9.6%)	27 (11%)
Prosthetic joint single stage revision	23 (9.2%)	22 (8.9%)
Closure with local or free flap[9]	45 (18%)	45 (19%)
New metalwork or implant placed[10]	47 (19%)	51 (21%)
Single Stage PJI	24 (9.6%)	22 (8.9%)
FRI with new internal fixation	23 (9.2%)	29 (11.7%)
Sinus present[11]	169 (68%)	167 (68%)
Purulence identified at surgery	102 (52%)	101 (53%)
Histopathology confirmatory for infection	133 (83%)	129 (79%)
Histology not recorded	88	84
Microbiology confirmatory for infection[12]	196 (79%)	175 (71%)
Organism identified		
Staphylococcus aureus	88 (35%)	84 (34%)
S. epidermidis	26 (10%)	27 (11%)
Streptococcus spp.	14 (5.6%)	18 (7.3%)
Enterococcus spp.	16 (6.4%)	12 (4.8%)
E. coli	11 (4.4%)	9 (3.6%)
Pseudomonas spp.	12 (4.8%)	10 (4.0%)
Polymicrobial infection	65 (26%)	52 (21%)

*All randomized patients except 3 who withdrew consent at randomization; n=497. Estimates and ranges are shown as median (IQR)

1: Gender unknown for 1 participant
2: Charlson Comorbidity Index unknown for 2 participants in Short antibiotic treatment group
3: BMI unknown for 4 participants in Long antibiotic group, 2 participants in Short group
4: Unknown whether vascular, neurological or renal complications of diabetes were present for 5 participants in Long antibiotic treatment group, 8 participants in short antibiotic treatment group
5: Smoking status unknown for 5 participants in Long antibiotic group, 3 participants in Short group
6: Unknown presence of peripheral vascular disease for 12 participants in Long antibiotic treatment group, 5 participants in Short group
7: Previous operation status unknown for 2 participants in Long antibiotic group, 5 participants in Short antibiotic group
8: Unknown operation for 1 participant in Short antimicrobial treatment group
9: Unknown means of surgical wound closure in 3 participants in Long antibiotic group, 6 participants in Short group
10: Unknown whether new implant placed for 1 participant in Short antibiotic group
11: Unknown whether sinus present for 1 participant in Short antibiotic group
12: Microbiology not recorded for 2 participants in Short antibiotic group

Supplementary Table S6 **Serious adverse events* by treatment arm** (excluding deaths and treatment failure)

**Shorter Arm** (n=234)		
**Event**	**Treatment**	**Required Hospital Admission**
Fracture Metatarsal	Unknown	N
Postop Haematoma	Return to theatre for drainage	Y
New allergy	Allergy recorded	N
Diarrhoea, hypokalaemia and deranged liver function tests	Stopped Itraconazole. Observation	N
Stevens-Johnson Syndrome	Stopped Doxycyline	N
Pin site infection	Oral antibiotics	N
Fall with soft tissue wrist injury	Rest, analgesia	N
Pulmonary fibrosis flare-up	Home oxygen and steroids	N
Diagnosis terminal oropharyngeal cancer	Palliative care	N
Elective surgery on elbow	Surgery	Y
Obstructed inguinal hernia	Surgery	Y
Suspected rigors	No treatment	Y
Cholecystitis	Surgery	Y
Psychosis	Psychiatric admission	Y
Recurrent UTI	Oral antibiotics	N
Oesophageal cancer	Radiotherapy and Chemotherapy	N
Severe UTI	IV antibiotics	Y
Wound breakdown at skin graft	Debridement and grafting	Y
Cholangitis	IV antibiotics	Y
Buccal biopsy	No treatment	Y
Paracetamol overdose	Observation	Y
Skin flap breakdown	Re-do graft	Y
Wound breakdown around flap	Revision surgery	Y
Cellulitis	Oral antibiotics	Y
Fall with fractured ulna	Internal fixation	Y
Pneumonia	IV antibiotics	Y
Fall, elbow fracture	Surgery	Y
Ankle fracture playing rugby	Surgery	Y
Intentional overdose	Observation	Y
Barrett's oesophagus with anaemia	Unknown	N
Heart failure	Unknown	N
Pressure ulcer	Debridement and antibiotics	Y
Knee subluxation	Manipulation under anaesthetic	Y
COVID 19	Dexamethasone	Y
Hepatitis C diagnosed	None	N
	**Total**	**35**
**Longer Arm** (n=241)		
Abdominal pain	Stopped clindamycin	Y
Tibia fracture at operative site	External fixation	Y
Non-union humerus fracture	Internal fixation	Y

Refracture of tibia	External fixation	Y
UTI	IV antibiotics	Y
Transaminitis	Stopped clindamycin	N
Drug rash	Steroid	N
Cholelithiasis/Cholangitis	IV antibiotics	Y
Multiple fractures in RTA	Surgery	Y
Fall soft tissue injury to foot	None	N
Collapse ?diagnosis	Observation	Y
Olecranon bursitis	IV antibiotics	Y
Fall with wrist fracture	Cast immobilisation	Y
New PJI knee (not index site)	Revision surgery	Y
Heel ulcer	Dressings	N
Post-op sepsis	IV antibiotics and ICU	Y
Stiff knee	Manipulation	N
Non-union femur fracture (not index site)	Surgery	Y
Abnormal electrolytes	Corrected	Y
Knee arthroscopy (not index site)	Surgery	Y
Haematoma	Observation	Y
Blocked percutaneous IV line	Line reinsertion	Y
Stiff knee	Manipulation	Y
Pneumonia	IV antibiotics	Y
Diarrhoea and Vomiting	IV fluids	Y
7th cervical nerve root entrapment	Unknown	N
Severe vomiting	Stopped antibiotics	Y
Collapse ?sepsis ?cardiogenic	IV antibiotics	Y
Fall fracture femur	Surgery	Y
Stroke with visual loss	Unknown	Y
Sepsis	Amputation	Y
Toe ulcer	Dressings and antibiotics	N
Hip dislocation	Relocated	Y
Fall, periprosthetic fracture	Surgery	Y
Severe nausea, anorexia, lethargy	IV fluids	Y
Cellulitis and foot ulcer	Additional antibiotics	Y
Wound breakdown	Surgery	Y
Acute kidney injury	Stopped antibiotics. Fluid support	Y
Arm fracture in RTA	Surgery	Y
	**Total**	**39**

*A serious adverse event was recorded if the event fulfilled at least one of the following criteria: Death (see Table S7), life threatening condition, required new or prolonged hospitalization, resulted in persistent or significant disability/incapacity.

Abbreviations: UTI, urinary tract infection; IV, intravenous; RTA, road traffic accident; PJI, periprosthetic joint infection; ICU, intensive care unit.

Supplementary Table S7 **Cause-specific Mortality by Treatment Arm with time to death**

**Shorter Arm** (n=234) 5 Deaths (2.1%)	
**Cause of Death**	**Time after Index Surgery (Days)**
Septic Shock due to *S. aureus* infection*	48
Hypoxic Cardiac Arrest	182
COVID 19	223
Liver Failure	347
Heart Failure, COVID 19	360
**Longer Arm** (n=241) 9 Deaths (3.7%)	
Acute myocardial infarction	5
Heart failure, failure of LVAD	31
Myocardial infarction	47
Frailty after Infected Femur Fracture*	100
Heart failure due to ischaemic heart disease	167
Aspiration Pneumonitis	189
Suicide	190
Suicide	199
Cardiac Arrythmia	260

Abbreviation: LVAD, Left ventricular assist device.

* Also recorded as treatment failure

REFERENCES

1 GBD 2021 Other Musculoskeletal Disorders Collaborators. Global, regional and national burden of other musculoskeletal disorders, 1990-2020, and projections to 2050: a systematic analysis of the Global Burden of Disease Study 2021. Lancet Rheumatol 2023; 5: e670-82

2 Gitajn IL, Werth PM, Carlini AR, et al. Deep Surgical Site Infection after Fracture Has a Profound Effect on Functional Outcomes. JBJS Open Access 2024;9(1):e23.00042.

3 Premkumar A, Kolin DA, Farley KX, et al. Projected Economic Burden of Periprosthetic Joint Infection of the Hip and Knee in the United States. The Journal of Arthroplasty 2021; 36(5):1484-1489.

4 Metsemakers W-J, Moriarty TF, Morgenstern M, et al. The global burden of fracture-related infection: can we do better? Lancet Infect Dis. 2023 https://doi.org/10.1016/S1473-3099(23)00503-0

5 Tissingh EK, Marais L, Loro A, et al. Management of fracture-related infection in low resource settings: how applicable are the current consensus guidelines? EFORT Open Rev 2022; 7(6): 422-432.

6 Hillier DI, Patrie MJ, Harrison TP et al. Financial analysis of revision hip surgery at a tertiary referral centre as classified using the British Hip

Society Revision Complexity Classification. Bone Jt Open 2023; 4-8: 559-566.

7 Kurtz S, Ong K, Lau E, Mowat F, Halpern M. Projections of primary and revision hip and knee arthroplasty in the United States from 2005 to 2030. J Bone Joint Surg Am. 2007;89-A(4):780–785.

8 Klug A, Gramlich Y, Rudert M, et al. The projected volume of primary and revision total knee arthroplasty will place an immense burden on future health care systems over the next 30 years. Knee Surg Sports Traumatol Arthrosc. 2021;29(10):3287–98.

9 Henriksen NL, Serrano-Chávez E, Fuglsang-Madsen A, et al. Gentamicin and clindamycin antibiotic-eluting depot technology eradicates S. aureus in an implant-associated osteomyelitis pig model without systemic antibiotics. Antimicrobiol Agents Chemo 2024; 68(10): 1-14.

10 Global burden of bacterial antimicrobial resistance 1990–2021: a systematic analysis with forecasts to 2050 GBD 2021 Antimicrobial Resistance Collaborators September 16, 2024 https://doi.org/10.1016/S0140-6736(24)01867-1

11 McNally MA, Sousa R, Wouthuyzen-Bakker M, et al. The EBJIS Definition of Prosthetic Joint Infection: a practical guide for clinicians. Bone Joint J 2021; 103-B(1): 18-25.

12 Govaert GAM, Kuhl R, Atkins BL, et al. Diagnosing Fracture-related Infections: current concepts and recommendations. J Orthop Trauma 2020; 34(1): 8-17.

13 Ravn C, Neyt J, Benito N, et al. Guideline for management of septic arthritis in native joints (SANJO). Journal of bone and joint infection. 2023 Jan 12;8(1):29-37.

14 Vach W (2013): Regression Models as a Tool in Medical Research. Chapman & Hall. Page 276-279.
